# Studies on the binding of CO to low-spin [Fe(II)(Por)L_2_] complexes: an aid to understanding the binding of CO to haemoglobin and myoglobin

**DOI:** 10.1007/s00775-022-01969-w

**Published:** 2022-12-07

**Authors:** Jack Silver, Golzar al-Jaff, Jehad A. Taies, Michael T. Wilson, Daniel den Engelsen, George R. Fern, Terry G. Ireland

**Affiliations:** 1 College of Engineering, Design and Physical Sciences, School of Engineering, Wolfson Centre for Materials Processing, Brunel University London, Kingston Lane, Uxbridge, UB8 3PH Middlesex UK; 2 School of Life Sciences, University of Essex, Wivenhoe Park, Colchester, CO4 3SQ Essex UK; 3 Department of Chemistry, College of Education, Salahaddin University-Erbil, Erbil, Iraq; 4 Department of Chemistry, College of Education for Pure Science, University of Anwar, Ramadi, Iraq

**Keywords:** Iron porphyrins, Mössbauer spectroscopy, Myoglobin, Haemoglobin, Carbon monoxide, Nitrogenous bases

## Abstract

The visible and Mössbauer spectra of [Fe(II)(Por)L_2_] and [Fe(II)(Por)L(CO)] complexes (where Por = protoporphyrin IX (PPIX) or tetra(*p*-sulfophenyl)porphyrin (TPPS) and L = an aliphatic or aromatic nitrogenous base) are reported and discussed. The results are compared to those of previously reported [Fe(II)(Por)L(CO)] complexes (where Por = PPIX, TPPS, PMXPP, TPP, OMTBP and OEP; L = a nitrogenous aromatic ligand) and HbCO (where Hb = haemoglobin) and MyCO (where My = myoglobin). A new approach, to extracting information from the Mössbauer parameters has been developed by plotting those of the [Fe(II)(Por)L_2_] complexes against those of [Fe(II)(Por)L(CO)] complexes for the same ligands, has yielded a series of trend lines that show a significant dependence on both the nature of the porphyrin and also of the nitrogenous ligand. Different trend lines were found for aromatic nitrogenous ligands to aliphatic nitrogenous ligands showing that the porphyrins could donate different amounts of charge to the Fe(II) cations as the L ligand changed, and hence, they display electron sink properties. From the plots, it was shown that haemoglobin and myoglobin both bind CO very strongly compared to the model complexes studied herein. Using the reported structural and Mössbauer data for the [Fe(II)(Por)L_2_] and [Fe(II)(Por)L(CO)] complexes, it proved possible and instructive to plot the Mössbauer parameters against a number of the bond lengths around the Fe(II) cations. The interpretation of the resulting trend lines both supported and facilitated the extension of our findings enabling further understanding of the geometry of the bonding in CO haemoglobin and CO myoglobin.

**Graphical abstract:**

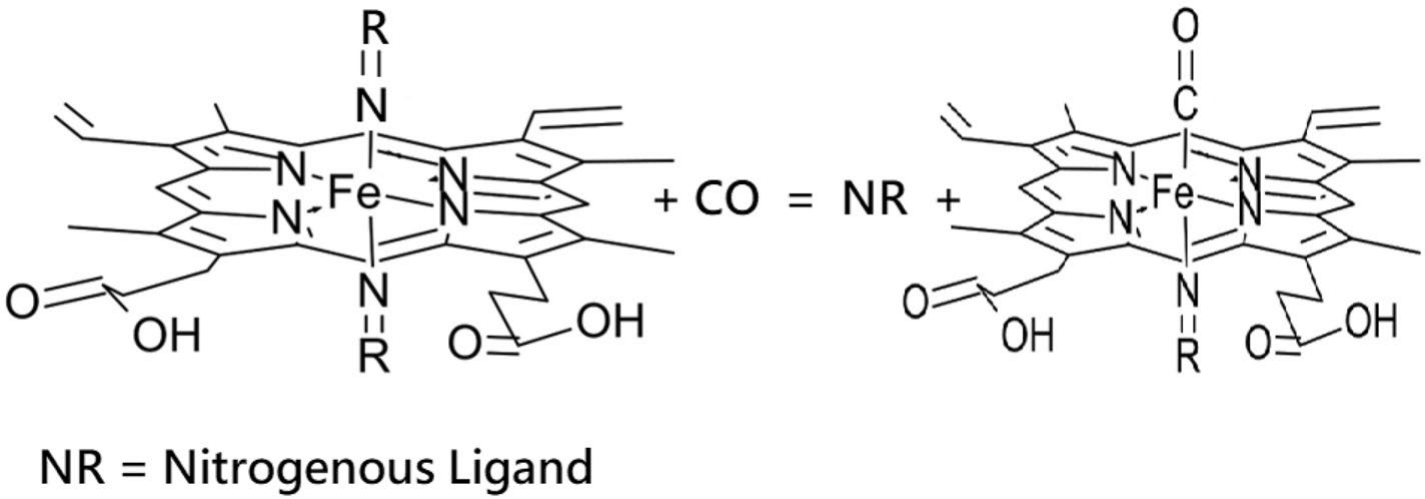

## Introduction

In natural enzymes, iron(II)protoporphyrin IX, [Fe(PPIX)] also known as haem b, is very widespread in nature [1–5]. [Fe(PPIX)] and related haems (iron porphyrin macrocycles), for example haem c and haem a [5]) are the active centres in a wide range of biological molecules each crucial for living organisms. The haem groups are used to perform a diversity of roles such as oxygen transport (haemoglobin) and storage (myoglobin), electron transport (the cytochromes) and in the elimination of toxic and unwanted compounds (cytochrome P_450_) [1–5]. In the last 25 years, a new group of enzymes (haemophores) haem transport proteins (used by bacteria and fungi to scavenge and transport haems from mainly animal sources) have been characterised [6]. The chemical properties of the iron in the haem are modulated/controlled both by the porphyrin and by the nature of the axial ligands [4, 5]. The manner in which the immediate environment of the iron atom is influenced by electron delocalisation on the macrocycle and the nature of the axial ligation in iron porphyrin complexes has been much discussed for over 5 decades [1–14]. Many structures/molecules containing natural and synthetic haems have been studied to obtain insight into porphyrin metal bonding interactions and how axial ligands may control and/or modify this bonding [15–20].

We have reported extensive studies on [Fe(PPIX)] chemistry using Mössbauer and optical spectroscopies [21–49]. We have demonstrated that by selecting the axial ligand, both the spin state of the [Fe(II)(PPIX)] and [Fe(III)(PPIX)) complexes and their geometry can be varied/controlled. In complementary studies, we have applied the understanding gained to a variety of biological molecules to begin to understand the chemistry carried out by these naturally occurring haems: (1) in haem peptides derived from cytochrome c [50–52]; (2) to the role of [Fe(PPIX)] in **P**orphyromonas gingivalis [47, 53–58] and other oral anaerobes [59, 60]; and (3) to haem-antimalarial complexes of pharmacological interest [61, 62].

There have been many studies on low-spin six-coordinate [Fe(II)(Por)L_2_] compounds (where Por = porphyrin and where L the axial ligands are nitrogenous aromatic or aliphatic ligands), that report crystallographic and/or Mössbauer spectroscopic data as we have previously reported [43, 44, 63]. A classic study on [Fe(II)(Por)L_2_] complexes that considered ligand orientation control gave amongst the major conclusions the fact that Mössbauer spectra provide a probe for ligand orientation when structural data may not be available [20]. This paper summarises and discusses their crystal structures comparing the relative orientation of the two axial planar ligands to each other and also to the four nitrogen atoms of the porphyrin core; in addition, it gives the Mössbauer parameters of the same complexes [20]. Unfortunately, there are fewer studies on the structures and Mössbauer spectra of [Fe(II)(Por)L(CO)] complexes [15–17, 27, 64–80]. There have in fact been only 18 Mössbauer spectra of [Fe(II)(Por)L(CO)] complexes reported in the literature, and moreover, they are a disparate group [17, 64–72]. In these complexes, only nine different L ligands have been reported, and these have been combined with seven different porphyrins (for Por = TPP (five complexes one reported twice), PMXPP (five complexes), TpivPP (one complex) OMTBP (two complexes) OEP (one complex) TPPS (one complex) and PPIX (three complexes). Of these complexes four have L = 1-MeIm, four more have L = piperidine, two have pyridine and two have glycine ethyl ester. The other five complexes each have a different L ligand. Therefore, although some generalisations on the bonding of the complexes have been made from the Mössbauer parameters, there is no consensus on the bonding in the complexes. This is even in the light of five crystal structures (four TPP and one OEP complexes) that had been studied by Mössbauer spectroscopy [68, 69, 76, 77, 79]. In addition, of the three complexes that contained PPIX (the porphyrin so widespread in nature) none contained an imidazole ligand [27, 66]. Therefore, no good models for natural systems have been studied. This is somewhat surprising as it is well established that the affinity between CO and haemoglobin (Hb) is around 200 times greater than that of oxygen with Hb [2, 3], and it might have been thought that model compounds of the type [Fe(II)(PPIX)L(CO)] would have been studied much more widely. Clearly a study of the latter complexes where the L ligands are a range of both aromatic and aliphatic nitrogenous ligands as well as where some of the L ligands have their binding properties sterically inhibited, would be very useful to gain insight into the properties that can affect the CO binding to the [Fe(II)(PPIX)L] moiety.

We have previously reported studies on a wide range of nitrogenous ligands binding to [Fe(II)(PPIX)] [43, 44, 63]. These studies covered the bonding of [Fe(II)(PPIX)] to pyridine, substituted pyridines, imidazole, aliphatic amines, piperidine and heterocyclic nitrogenous bases. The results were compared to previous literature on binding studies of pyridine and imidazole’s to haems in non-aqueous systems and in so doing we summarised the many different factors that affect such binding. In our approach we used plots of the p*K*_a_ value of the ligand against its log*β*_2_ value and of Δ*E*_Q_ (its Mössbauer quadrupole splitting parameter) against log*β*_2_ value to analyse the results, where *β*_2_ is the overall equilibrium constant of the reaction between [Fe(II)(PPIX)] and a nitrogenous ligand. This then innovative combination/display of the data was used to underpin our conclusions [43, 44, 63]. It allowed us to compare the nitrogenous bases and the way they bind to [Fe(II)(PPIX)] in detail. We will refer to some of these results in this work where we discuss our studies on [Fe(II)((PPIX)L(CO)] complexes.

Herein, we report the preparation of new low-spin six-coordinate [Fe(II)(PPIX)L(CO)] complexes where L is either an aliphatic or aromatic nitrogenous ligand. Some of the aromatic ligands contain two or more *N* atoms or another hetero atom in the aromatic ring. To gain further insight into how variation in the bonding properties of such ligands can affect the [Fe(II)(PPIX)] entity in the complexes when CO is present as the sixth ligand, we have studied both their electronic absorption spectra and Mössbauer spectra (obtained from frozen solutions of the complexes). These investigations were directed at examining *σ*- and *π*-bonding effects as well as steric effects in the bonding of the axial ligands in the presence of CO. Protoporphyrin IX was the porphyrin selected for the studies because it is the most widespread porphyrin found in natural proteins. Other reasons for its selection and its limitations have previously been discussed [37–42]. Following on from those studies our investigations were carried out at high pH (above 11.95) where we have previously shown that a significant proportion of the [Fe(II)(PPIX)] is monomeric in the absence of nitrogenous bases. At lower pH values (7.7–11.0), the aggregated [Fe(II)(PPIX)] are the dominant species in solution and such species complicate ligand-binding studies [24, 32].

As the [Fe(II)(PPIX)L(CO)] complexes were water soluble, we felt that it was useful to gain further insight into the solution chemistry of these complexes, we set out to compare and contrast it to the chemistry of tetra(*p*-sulfophenyl)porphyrin iron(II), [Fe(TPPS)], which is another water-soluble porphyrin we have studied previously [64, 81–83]. We, therefore, report both the electronic absorption spectra and Mössbauer spectra of [Fe(TPPS)L(CO)] complexes (also obtained from frozen solutions of the complexes).

## Experimental

Haematin was purchased from Sigma and used without further purification. The nitrogen ligands (presented in Tables 1 and 2) were either purchased from Aldrich or supplied by ICI. The extremely sterically hindered ligands: tert-butylamine (from Aldrich) was distilled from KOH prior to spectral use: 2-methylpyridine (from Aldrich) was fractionally distilled from KOH.

**Table 1:** The electronic absorption spectra (recorded at 25 °C) of the low-spin [Fe(PPIX)L(CO)] and [Fe(PPIX)L_2_] complexes (where L = nitrogenous ligand) at pH 12, *λ*_max_ show Soret, *β* and *α* bands

Nitrogenous ligand	Band maxima (nm)			References
	Soret band	*β* band	*α* band	
[Fe(II)(PPIX)L(CO)] complexes				
4(3) Pyrimidone	410	543	573	^c^
2-Methylpyrazine	412	543	568	^c^
2-Methoxypyrazine	415	533	562	^c^
Thiazole	413	540	564	^c^
Oxazole	411	538	566	^c^
4-Amino 1,2,4-triazole	412	538	565	^c^
Imidazole	413	537	567	^c^
5-Chloro-1-methylimidazole	415	541	563	^c^
2-Methylimidazole^a^	416	538	566	^c^
Tertiarybutylamine^a^	415	535	568	^c^
2-Methylpyridine	413	544	574	^c^
Pyridine-*N*-oxide^a^	415	541	563	^c^
Cysteine methyl ester	416	536	567	[27]
Glycine ethyl ester	416	536	566	[27]
[Fe(II)(PPIX)L_2_] complexes				
4(3)Pyrimidone	418	523	554	[63]
2-Methylpyrazine	421	530	564	[63]
2-Methoxypyrazine	419	530	564	[63]
Thiazole	418	525	558	[63]
Oxazole	418	529	558	[63]
4-Amino 1,2,4-triazole	415	524	556	[63]
Imidazole	421	526	555	[43]
5-Chloro-1-methylimidazole	421	525	558	[43]
2-Methylimidazole^b^	425	–	556	[63]
Tertiarybutylamine^b^	422	–	557	[63]
2-methylpyridine	418	528	562	[63]
Pyridine-*N*-oxide^b^	418	–	556	[43]
Cysteine methyl ester	419	524	556	[27]
Glycine ethyl ester	421	525	555	[27]

^a^These three complexes are six coordinates in the presence of CO

^b^These three complexes have typical spectra of high spin five coordinate iron(II) porphyrin complexes. However, at liquid nitrogen temperature, they all form some low-spin six-coordinate [Fe(PPIX)L_2_] complex (see Ref. [63])

^c^This work

**Table 2:** The electronic absorption spectra (recorded at 25 °C) of the low-spin [Fe(TPPS)L(CO)] and [Fe(TPPS)L_2_] complexes (where L = nitrogenous ligand) at pH 12, *λ*_max_ show Soret, *β* and *α* bands

Nitrogenous ligand	Band maxima (nm)			References
	Soret band	*β* band	*α* band	
[Fe(TPPS)L(CO)] complex where L =				
Pyridine	421	535	580^a^	^b^
2-Methyl pyridine	422	541	580^a^	^b^
4-Methyl pyridine	423	540	580^a^	^b^
3,4-Dimethyl pyridine	424	537	580^a^	^b^
3-Amino methyl pyridine	423	543	580^a^	^b^
Imidazole	424	540	580^a^	^b^
4-Amino-1,2,4-triazole	420	543	580^a^	^b^
*n*-Butyl amine	423	541	580^a^	^b^
3-Aminopropionic acid	422	540	580^a^	^b^
Piperidine	423	541	580^a^	^b^
Pyrrolidine	423	544	580^a^	^b^
Glycine ethyl ester	420.7	540.8	580^a^	[64]
[Fe(TPPS)L_2_] complex where L =				
Pyridine	424	529	562	^b^
2-Methyl pyridine	428	532	564	^b^
4-Methyl pyridine	425	531	562	^b^
3,4-Dimethyl pyridine	427	532	564	^b^
3-Amino methyl pyridine	425	531	563	^b^
Imidazole	427	533	566	^b^
4-Amino-1,2,4-triazole	425	532	564	^b^
*n*-Butyl amine	428	533	562	^b^
3-Aminopropionic acid	427	533	565	^b^
Piperidine	428	532	562	^b^
Pyrrolidine	428	533	565	^b^
Glycine methyl ester	423	532	563	[64]

^a^A very small absorption band around 580 nm difficult to define position better

^b^This work

Haematin was first dissolved in NaOH (0.1 M) and then diluted to the desired concentration (~ 10^–5^ M) with NaOH to give a solution of final pH = 12. The haematin was reduced to PPXIFe(II) with a slight excess of solid sodium dithionite.

Tetra(*p*-sulfophenyl)porphyriniron(II) solutions were prepared as previously reported [64, 82].

Electronic absorption spectra were obtained using a DU-7 spectrophotometer (Beckman) between 350 and 750 nm.

Mössbauer spectra were recorded on concentrated frozen solutions at 78 K. The Mössbauer spectrometer and experimental details have previously been described [65].

## Results and discussion

In this work, 14 nitrogenous ligands were studied with CO and [Fe(PPIX)]; the absorption peaks in the visible region of the resulting [Fe(PPIX)L(CO)] complexes together with those of the related [Fe(PPIX)L_2_] complexes are presented in Table 1. In Table 2, we report the absorption peaks in the visible region of [Fe(TPPS)L(CO)] complexes together with those of the related [Fe(TPPS)L_2_] complexes of 2 nitrogenous ligands. All ligand names used in this paper are given in full in Tables 1 and 2). The abbreviations used for the nitrogenous ligands appear all together in Tables 3 and 4, and those used for the porphyrins are in Tables 4 and 5 and their footnotes.

**Table 3:** 57Fe Mössbauer spectral data for low-spin [Fe(Por)L(CO)] complexes (where L = nitrogenous ligand)

Complex	*T* °K	*δ* (mm s^−1^)^a^	Δ*E*_Q_ (mm s^−1^)^b^	Г (mm s^−1^)^c^	References
1. [Fe(PPIX)(EtNH_2_)(CO))]	77	0.26(1)	0.33(1)	0.14(2)	^f^
2. [Fe(PPIX)((CH_2_NH_2_)_2_)(CO)]	77	0.33(1)	0.41(1)	0.21(2)	^f^
3. [Fe(PPIX)(butNH_2_)(CO)]	77	0.26(1)	0.38(1)	0.14(1)	^f^
4. [Fe(PPIX)(^s^buNH_2_)(CO)]	77	0.23(1)	0.39(1)	0.15(1)	^f^
5. [Fe(PPIX)(pip)(CO)]	77	0.26(1)	0.57(2)	0.13(1)	^f^
6. [Fe(PPIX)(4-Mepy)(CO)]	77	0.33(1)	0.58(1)	0.19(1)	^f^
7. [Fe(PPIX)(Im)(CO)]	77	0.26(1)	0.27(1)	0.15(1)	^f^
8. [Fe(PPIX)(4-NHTRIZ)(CO)]^d^	77	0.28(1)	0.43(2)	0.18(1)	^f^
9. [Fe(PPIX)(4-*n*-butTRIZ)(CO)]^d^	77	0.29(2)	0.43(3)	0.18(2)	^f^
10. [Fe(PPIX)(Me(TTZProp)(CO)]^d^	77	0.28(1)	0.61(1)	0.13(1)	^f^
11. [Fe(PPIX)(2-MeIm)(CO)]	77	0.22(1)	0.36(2)	0.19(2)	^f^
12. [Fe(PPIX)(tbutNH2)(CO)]^e^	77	0.36(1)	0.57(1)	0.18(1)	^f^
13. [Fe(PPIX)(2-Mepy)(CO)]	77	0.26(1)	0.59(2)	0.23(2)	^f^
14. [Fe(PPIX)(GEE)(CO)]	80	0.27(2)	0.42(2)	0.14(2)	[27]
15. [Fe(PPIX)(CME)(CO)]	80	0.26(2)	0.49(2)	0.15(2)	[27]
16. [Fe(PPIX)(pip)(CO)]	115	0.26	0.57	0.14	[66]
17. [Fe(TPPS)(pip)(CO)]	78	0.32(1)	0.57(1)	0.20(1)	^f^
18. [Fe(TPPS)(pydn)(CO)]	78	0.31(2)	0.42(4)	0.15(6)	^f^
19. [Fe(TPPS)(py)(CO)]	78	0.28(1)	0.56(1)	0.16(1)	^f^
20. [Fe(TPPS)(4-Mepy)(CO)]	78	0.27(1)	0.49(2)	0.19(2)	^f^
21. [Fe(TPPS)(3,4 Me2py)(CO)]	78	0.28(3)	0.57(1)	0.30(4)	^f^
22. [Fe(TPPS)(3MeNHpy)(CO)]	78	0.30(1)	0.43(2)	0.23(3)	^f^
23. [Fe(TPPS)(GEE)(CO)]	78	0.28(1)	0.37(1)	0.19(1)	[64]

Ligand names abbreviated herein are given in full in Tables 1 and 2

^a^δ (The isomer shift) is relative to iron foil

^b^Δ*E*_Q_ is the Mössbauer quadrupole splitting

^c^Half width at half height. Where 4-NHTRIZ = 4-*n*-butyl 1,2,4-triazole; 4-*n*-butTRIZ = 4-*n*-butyl 1,2,4-triazole; and Me(TTZProp) = Methyl 2-(1,2,3,4-tetrazol-3-yi)-propionate

^d^These spectra contained a second species which had the parameters of the {Fe(PPIX)L_2_] complex as seen in Table 4

^e^This spectrum gave evidence for 45 (2)% of this low-spin complex and 23 (3)% of a five coordinate high spin complex believed to be [Fe(PPIX)(^t^ButNH_2_)] and 32 (2)% of the unreacted (none ligated to ^t^ButNH_2_) starting [Fe(PPIX)]

^f^This work

**Table 4:** 57Fe Mössbauer data for other low-spin [Fe(Por)L(CO)] complexes (where L = nitrogenous ligand)

Compound	*T* °K	*δ* (mm s^−1^)	Δ*E*_Q_ (mm s^−1^)	References
[Fe(TPP)(1-MeIm)(CO)]	77	0.20^a^	0.35	[68, 69]
[Fe(TPP)(py)(CO)]	77	0.28^a^	0.57	[68, 69]
[Fe(TPP)(pip)(CO)]	115	0.25	0.47	[66]
[Fe(TPP)(1-MeIm)(CO)]^b^	293	0.16	0.35	[69]
	100	0.25	0.32	[69]
[Fe(TPP)(1,2-Me_2_Im)(CO)]^b^	293	0.17	0.71	[69]
	100	0.29	0.66	[69]
[Fe(PMXPP)(Morph)(CO)]	298	0.22	0.55	[70]
[Fe(PMXPP)(pip)(CO)]	298	0.20	0.49	[70]
[Fe(PMXPP)(py)(CO)]	298	0.19	0.49	[70]
[Fe(PMXPP)(Pydn)(CO)]	298	0.18	0.45	[70]
[Fe(PMXPP)(Im)(CO)]	298	0.18	0.36	[70]
[Fe(TpivPP)(1-MeIm)(CO)]	4.2	0.27	0.27	[71, 72]
[Fe(OMTBP)(pip)(CO)]	115	0.30	0.20	[66]
[Fe(OMTBP)(1-MeIm)(CO)]	115	0.29	0.0	[66, 67]
[Fe(OEP)(1-MeIm)(CO)]^b^	293	0.18	0.40	[69]
	100	0.23	0.37	[69]
MbCO	4.2	0.20	0.35	[15, 16, 73]
HbCO	4.2	0.19	0.36	[17, 74]
CytochromeP450_cam_CO	200	0.16	0.34	[75]
[Fe(pc)(py)(CO)]	295	0.11	1.19	[76]
[Fe(pc)(pip)(CO)]	295	0.11	1.27	[66, 76]
[Fe(pc)(NH_3_)(CO)]	295	0.12	1.02	[76]
[Fe(pc)(n-prNH_2_)(CO)]	295	0.10	1.11	[76]

Ligand names abbreviated herein are given in full in Tables 1 and 2

^a^δ value not given in Ref. 68 but estimated from spectra accuracy not high

^b^Crystal structure contains a solvent molecule

Macrocyclic ligands appearing in the above complexes are defined: TPP = α, β, γ, δ–tetraphenylporphyrin dianion; PMXPP = tetra(*p*-methoxyphenyl)porphin dianion, TpivPP = “Picket Fence”porphyrin dianion = tetra(*o*-pivalamidophenyl)porphyrin dianion; OMTBP = octamethyltetrabenzoporphyrin dianion; OEP = octaethylporphyrin dianion, pc = phthalocyanine dianion

**Table 5:** Crystal structure data for low-spin [Fe(Por)L(CO)] complexes and [Fe(Por)L_2_] complexes

Compound	N_ax_–Fe Å	N_Por_–Fe Å	Fe–C Å	C–O Å	Total of six bond lengths (Å)	References
[Fe(TPP)(pip)_2_]	2.127(3)	2.004(6)			12.270	[84]
[Fe(TPP)(py)_2_]	2.037(1)	2.001(2)			12.082	[85, 86]
[Fe(TPP)(1-VinylIm)_2_]	2.004(2)	2.001(2)			12.012	[87, 88]
[Fe(TPP)(1-BzylIm)_2_]	2.017(4)	1.993(9)			12.040	[87]
[Fe(TPP)(1-MeIm)_2_]	2.014(5)	1.997(6)			12.044	[87]
[Fe(TPP)(py)(CO)]	2.10(1)	2.02(3)	1.77(2)	1.12(2)	11.95	[67, 75]
[Fe(TPP)(1-MeIm)(CO)]	2.071(2)	2.003(5)	1.793(3)	1.061(3)	11.875	[76]
[Fe(TPP)(1-MeIm)(CO)].C_6_H_6_	2.0503(14)	2.005(6)	1.7600(17)	1.139(2)	11.8308	[68]
[Fe(TPP)(1,2-Me _2_Im)(CO)].C_7_H_8_	2.0779(11)	1.985(8)	1.7537(15)	1.1408(19)	11.7716	[68]
[Fe(TFPP)(Fe(C_5_H_5_)(C_4_H_4_N))_2_]	2.05(2)	2.01(2)			12.14	[89]
[Fe(TpivPP)(1-MeIm)_2_]	1.9958(19)	1992(3)			11.9595	[90]
[Fe(TpivPP)(1-EtIm)_2_]	2.0244(18)	1.993(6)			12.0208	[90]
[Fe(TpivPP)(1-VinylIm)_2_]	1.9979(19)	1.988(5)			11.9478	[90]
[Fe(Tpiv_2_C_12_P)(1-MeIm)(CO)]^a^	2.062(5)	1.999(3)	1.728(6)	1.149(6)	11.786	[91]
[Fe(II)(OEP)(1-MeIm)(CO)]	2.077(3)	2.000(3)	1.744(5)	1.158(5)	11.821	[68, 78]
[Fe(TMP)(4-CNpy)_2_]	1.996(2)	1.993(2)			11.97	[92]
[Fe(TMP)(4-Mepy)_2_]	2.010(2)	1.988(2)			12.016	[92]
[Fe(TMP)(2-MeHIm)_2_]	2.047(3) 2.030(3)	1.964(5)			12.028	[20]
[Fe(TMP)(2-MeHIm)_2_]	2.032(3) 2.028(3)	1.961(7)			12.042	[20]

TMP = *meso*-tetramesitylporphyrin dianion, Tpiv_2_C_12_P = 5,15-[2,2-(dodecanediamido)-diphenyl]:α, α-10,20-bis(*o*-pivaloylaminophenyl)porphyrin dianion

^a^Crystal structural data are for this complex, but Mössbauer data are for the [Fe(TpivPP)(1-MeIm)(CO)] complex

### Visible spectra

Contemplating the known iron(II)porphyrin crystal structures [6–19], a useful observation can be made, that is: “the porphyrin ring is essentially a plane and the iron atom in it has D_4h_ symmetry” [93]. The spectral bands in the visible region arise from the extensive delocalisation of π electrons on the porphyrin and we have discussed such spectra in detail as have others [94]. Those reported herein for the [Fe(Por)L_2_] complexes are similar to those we previously reported [43, 44, 63], and in these studies and those reported by others [94], it was found that the Soret band of porphyrin iron(II) complexes coordinated to aliphatic ligands shifts to longer wavelengths while with unsaturated ligands (*π*-bonded systems) the Soret band moves towards shorter wavelengths. Simply put the explanation of the movement of the Soret band is that when *π*-electron charge from the metal atom *t*_2g_ orbitals moves outwards towards the periphery of the porphyrin, absorption occurs at longer wavelengths. Therefore, as electron charge is donated to the iron by/from the saturated ligands, it is accumulated in the *z* direction; this will only have a slight effect on the spectrum (not affecting the *π* electrons of the porphyrin nitrogen atoms in the *xy* plane) [95]. In contrast when unsaturated ligands bind to the iron the metal *t*_2g_ orbitals (*d*_*yz*_, *d*_*xz*_) are involved in *π*-bonding to them. This results in a decrease in the overlap of metal *t*_2g_ orbitals with the *π* orbitals of the porphyrin ring (via the porphyrin nitrogen atoms) leading to the shift of the Soret band to shorter wavelengths [43, 44, 63, 93–96].

**Fig. 1:**
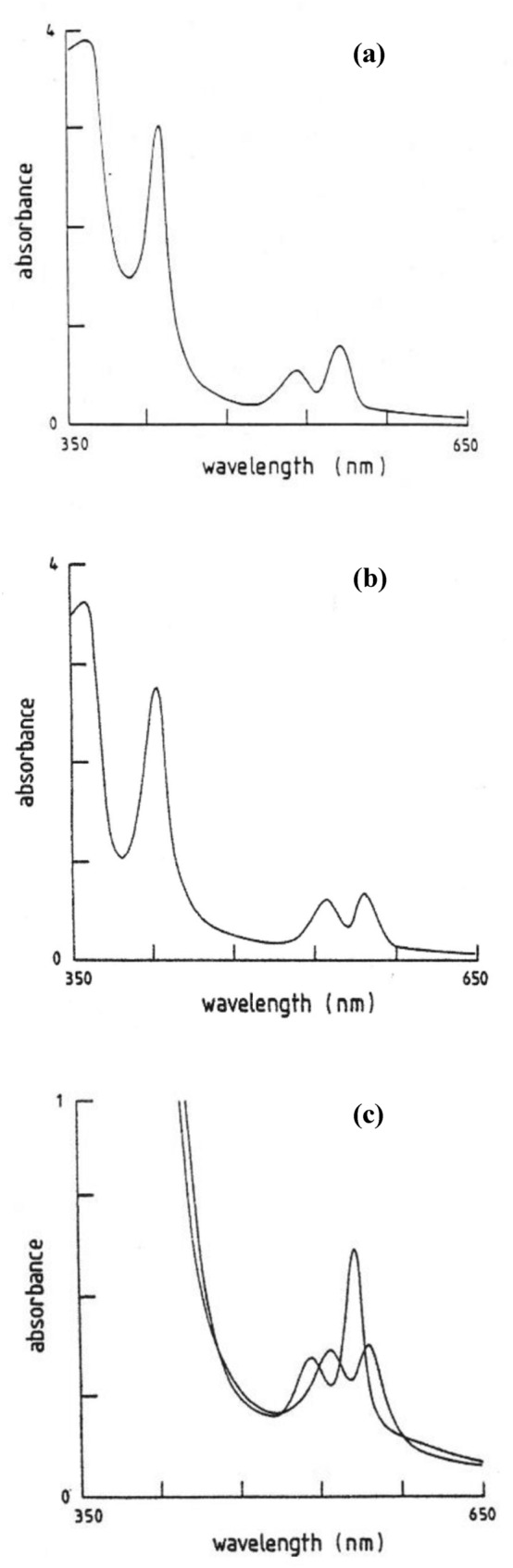
The visible spectra of **a** [Fe(II)(PPIX)(Im)_2_] and **b** [Fe(II)(PPIX)(Im)(CO)] at pH 12.0. The concentration of the [Fe(II)(PPIX)] solution used was 2 × 10^–5^ M. The overlay of the spectra in (**a**) and (**b**) are magnified in (**c**) to show the movement of the β band on forming the CO complex (the markers on the wavelength axis are at 60 nm intervals)

In Table 1, the spectra [i.e. the peaks of the absorption bands] of the low-spin [Fe(PPIX)L(CO)] and [Fe(PPIX)L_2_] complexes (L = nitrogenous ligand) studied are presented. A typical set of spectra are presented in Fig. 1 (for L = Im). Clearly, in the visible spectra of the [Fe(PPIX)L(CO)] complexes when compared to the spectra of the pure [Fe(PPIX)L_2_], the Soret bands are shifted to shorter wavelength (by between 3 and 9 nm). This is said to be due to the balance between the relative *σ*-donor and *π*-acceptor character of these ligands when one CO is bound to the haemachrome. The CO will even replace a strongly bound nitrogenous ligand, for example imidazole and there will then be a competition between these two ligands for the sixth position. Alben and Caughey [17] have explained the above observation by suggesting that the greater the basicity (the *σ*-donor strength of these ligands), then the easier it is to replace the sixth ligand by CO (a strong *π*-acceptor); this is a trans-effect.

It should be noted that the two [Fe(PPIX)L_2_] complexes where L is imidazole or 5-chloro-1-methylimidazole ligand have Soret bands more like those expected for unsaturated ligands, we commented on this previously [43], and suggested that imidazole has better *σ*-donating properties than the other aromatic ligands. In contrast the *β* band moves to longer wavelength in the [Fe(PPIX)L(CO)] complexes when compared to the spectra of the pure [Fe(PPIX)L_2_]: this movement varies between 11 and 20 nm (again see Fig. 1 for L = Im).

Three of the complexes in Table 1 (the pyridine-*N-*oxide, the 2-methyl imidazole and the Tert-butylamine) do not form low-spin {Fe(PPIX)L_2_] complexes due to the steric hindrance of the ligands and in fact form typical high spin five coordinate [Fe(PPIX)L] complexes as identified by the spectra in Table 1. But these complexes change to low-spin six-coordinate [Fe(PPIX)L(CO)] complexes when CO binds to them.

**Fig. 2:**
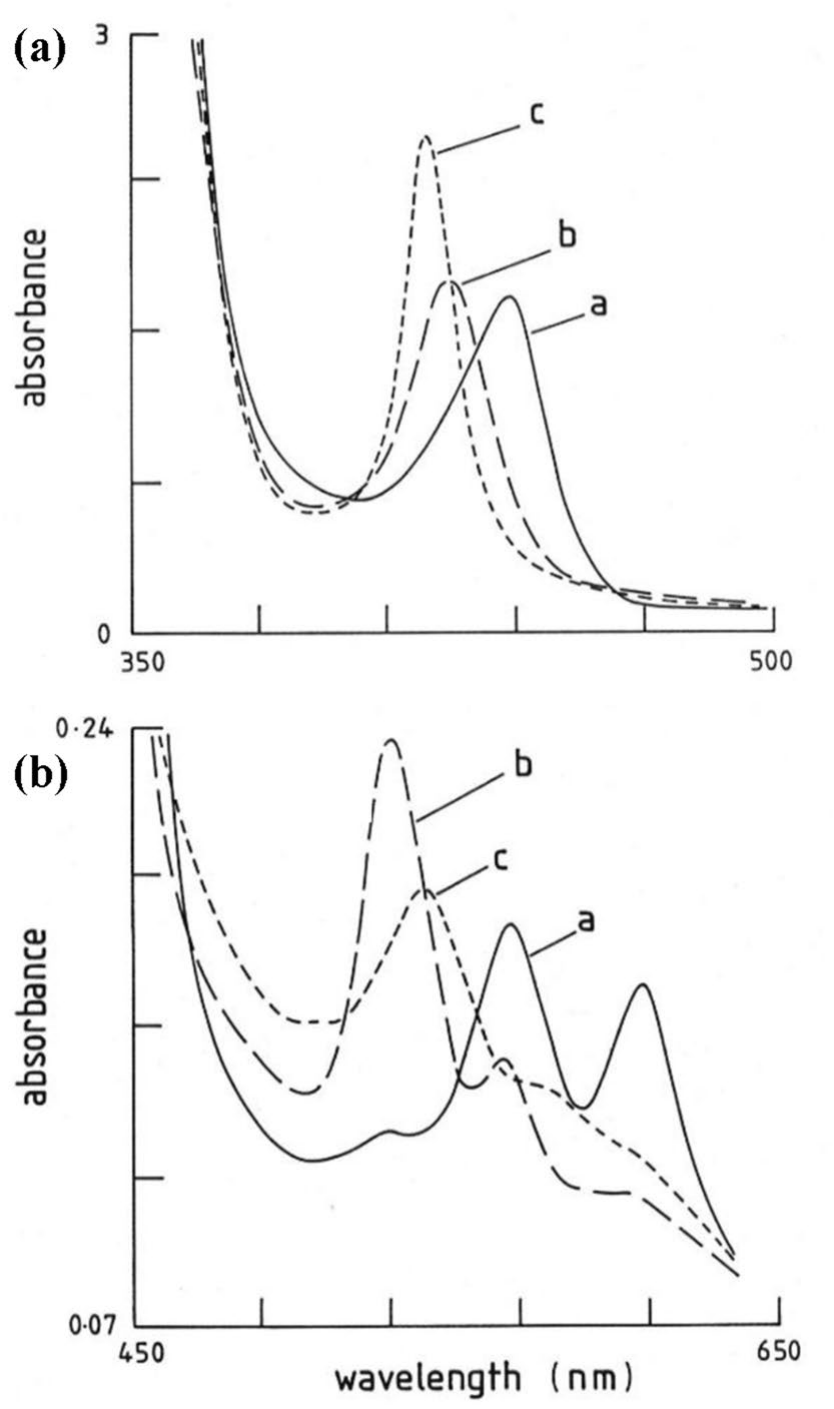
**a** The Soret bands and **b** the α and *β* bands of (a) [Fe(II)(TPPS)] in aqueous solution at pH 12.0; (b) [Fe(II)(TPPS)(n-butylamine)_2_] and (c) [Fe(II)(TPPS)(n-butylamine)(CO)] complexes in solution at high pH. The concentration of the [Fe(II)(TPPS)] solution used was 5 × 10^–5^ M. (The markers on the wavelength axis are at intervals of 30 nm (**a**) and 40 nm (**b**) from each other)

The electronic absorption spectra of the [Fe(TPPS)L(CO)] and [Fe(TPPS)L_2_] complexes are presented in Table 2, and in Fig. 2, spectra are presented for the L = *n*-butylamine complexes. It is apparent that the Soret bands of both sets of complexes are at longer wavelengths than those of the complexes in Table 1. This is evidence that the *π*-electron cloud on TPPS is more focussed towards the Fe(II) atom than is the case for PPIX. This may be a result of the four periphery negative charges on the sulphate ions on the TPPS phenyl rings repelling the porphyrin *π*-cloud in the direction of the iron(II). The fact that Fe(II)TPPS behaves differently to Fe(II)PPIX is in keeping with our previous work on these and other water-soluble Fe(II)Porphyrins in frozen solution [23, 26, 32, 81–83, 97, 98]. Indeed, the species present in solution at high pH are similar though the proportions are different and there is more aggregated species in Fe(II)TPPS solution at pH 12 and above (see Fig. 2) and that is why the Soret band becomes much sharper and more intense when this species is converted to the [Fe(II)(TPPS)L_2_] and [Fe(II)(TPPS)L(CO)] complexes. The Soret bands of the [Fe(II)(PPIX)L_2_] complexes all lie in the range 415–421 nm, and on forming the [Fe(II)(PPIX)L(CO)] complexes, the new range is from 410 to 415 nm. In contrast, the Soret bands for the [Fe(II)TPPS)L_2_] complexes are in the range 424–428 nm, while the [Fe(II)(TPPS)L(CO)] complexes range from 420 to 424 nm.

The *β* bands of the [Fe(II)(PPIX)L_2_] complexes are in the range 523–530 nm, and on forming the [Fe(II)(PPIX)L(CO)] complexes, the new range is from 533 to 544 nm. In the case of the [Fe(II)TPPS)L_2_] complexes, the *β* bands are in the range 529–533 nm, while for the [Fe(II)(TPPS)L(CO)] complexes range from 535 to 544 nm, in addition, the latter bands are very intense. The largest movement in these bands takes place for the [Fe(II)(PPIX)L(C)O)] complexes.

The behaviour of the *α* bands is very different for the [Fe(II)(PPIX)L_2_] complexes and the [Fe(II)(PPIX)L(CO)] complexes compared to those for the [Fe(II)TPPS)L_2_] complexes and the [Fe(II)(TPPS)L(CO)] complexes. The *α* bands for the [Fe(II)(PPIX)L_2_] complexes are in the range 555–564 nm and those for [Fe(II)(PPIX)L(CO)] complexes are in the range 562–574 nm. In total contrast, the bands in the [Fe(II)TPPS)L_2_] complexes are in a tight range from 562 to 566 nm while those for the [Fe(II)(TPPS)L(CO)] complexes have very small intensity and are around 580 nm. From these results, it appears that the porphyrin *π*-electron density in the [Fe(II)(TPPS)L(CO)] complexes are concentrated more towards the iron(II) than in the [Fe(II)(PPIX)L(CO)] complexes. We will present more evidence in support of this conclusion in the next section.

It is apparent from Tables 1 and 2 that all the spectra change when an axial nitrogenous ligand is exchanged for a CO molecule, and in all the cases, the Soret band moves to shorter wavelength and the *β* and α bands move to longer wavelength. This is evidence that the energy levels of the porphyrin orbitals are modified irrespective of whether the remaining nitrogenous axial ligand is aliphatic or aromatic; hence, in each case, the highest occupied porphyrin ring orbitals need to donate more electron density to the iron(II) atom to compensate for the *π*-back-bonding requirements of the CO ligand. The fact that the spectra vary with the different nitrogenous ligands is evidence for the electron sink properties of the porphyrin moieties. More evidence in support of this is manifest in the Mössbauer data and is discussed as follows.

It is also apparent that the porphyrin spectral changes observed are not just due to changes in the axial ligand bonding, (i.e. not just due to the amount of *σ*- and *π*-bonding the ligands donate/remove to/from the iron that modifies the spectra;) as any steric effects interfering with the ligands bonding will have a role, as will the interactions of the iron(II)porphyrins with solvent molecules. In addition, the interactions of solvent molecules with the iron(II) porphyrins may be modified by the varying properties of the different nitrogenous ligands. Thus, many factors will affect the positions of the porphyrin bands in addition to bonding to the iron(II) ion. However, from the point of view of the iron(II) ion, the bonding orbitals of the axial ligands and the porphyrin will be the active entities in modifying its bonding, and Mössbauer spectroscopy is a useful probe for this, as will be discussed in the next section.

### Mössbauer spectroscopy

To aid in the understanding of the electronic environments around the iron(II) centres and how these are affected by the binding of ligands, Mössbauer spectra were collected on frozen solutions at 78 K. The spectra of all the [Fe(II)(Por)L(CO)] (where Por = PPIX and TPPS) complexes consisted of sharp doublets and the parameters are presented in Table 3. The spectral data of the other [Fe(II)(Por)L(CO)] complexes found in the literature are given in Table 4 for comparison. [The Mössbauer parameters listed in Tables 3 and 4, viz. *δ*, Δ*E*_Q_ and are defined, in the legends of Table 3].

It is also important to bear in mind when recording Mössbauer parameters from six-coordinate low-spin iron(II) porphyrin complexes (and also six-coordinate low-spin iron(III) porphyrin complexes) in frozen solutions that the nature of the solution and the nature of the substituent groups on the axial ligands can affect the bonding of the axial ligands to the porphyrin [34, 35, 37–42]. This can result for instance in the Δ*E*_Q_ values of the complex varying with the pH of the solution before it was frozen or varying with the solution itself (whether it was aqueous or non-aqueous). Such different solutions can affect the hydrogen bonding to and around the axial ligand. For ligands such as histidine and imidazole, changes in the hydrogen bonding can lead to changes in the orientation of the planes of the two axial ligands to each other and also to their orientation with the porphyrin nitrogen to iron bonds [39, 40]. We have discussed such effects in detail previously [34–42]. It is obvious from this discussion that any changes in the solvent can also affect the orientation of both conjugated and non-conjugated substituent groups on both aliphatic and aromatic nitrogenous ligands. This is also relevant to the [Fe(II)(Por)(L)(CO)] complexes discussed below as their nitrogenous axial ligands can also be influenced in the same way by the surrounding environment.

The Mössbauer parameters for the [Fe(II)(PPIX)L)-(CO)] complexes are presented in Table 3. The isomer shifts (all referenced to natural iron foil) of these complexes have an average value of 0.27 mm s^−1^ and are reduced by about 0.2 mm s^−1^ from those of the corresponding [Fe(PPIX)(L_2_)] complexes, whilst the Δ*E*_Q_ values range between 0.27 and 0.61 mm s^−1^ (reduced by around 0.6 mm s^−1^ from the [Fe(II)(PPIX)L_2_] complexes) [44]. The exceptions are the results with ligands such as triazole and tetrazole spectra 8–10 of Table 3 and also spectrum 12. The frozen solution of these complexes proved to be a mixture of the low-spin [Fe(PPIX)(L_2_)] and the[Fe(PPIX)(-L)-(CO)] complexes. The [Fe(PPIX)(-L)-(CO)] complexes, where L is a sterically hindered ligand (see complexes 11–13 Table 3), readily form when CO is bubbled into the parent solutions; total conversion was found for complexes 11 and 13, whereas in the case of complex, 12 42% of the [Fe(PPIX)] present was in the form of the carbonyl complex which was more than double the amount of low-spin complex than before the addition of the CO. The results on these three sterically hindered ligand complexes show that the presence of the CO stabilises/reinforces the bonding of sterically hindered ligands. The [Fe(PPIX)(L)(CO)] complexes are characterised by low isomer shifts and Δ*E*_Q_ values; however, the overall magnitude of the Δ*E*_Q_ though dominated by the contribution from the CO appears to be affected by the bonding properties of the axial ligand as well.

In contrast to the [Fe(II)(PPIX)L(CO)] complexes, the isomer shift range found for the [Fe(II)(TPPS)L(CO)] complexes (complexes 17–23 also given in Table 3) were between 0.14 and 0.18 mm s^−1^; less than those of the corresponding [Fe(II)(TPPS)L_2_] complexes [64, 99] and that for the Δ*E*_Q_ values is between 0.37 and 0.57 mm s^−1^. The average isomer shift for the [Fe(II)(PPIX)L(CO)] complexes is 0.27 mm s^−1^ whereas that for the [Fe(II)(TPPS)L(CO)] complexes is 0.29 mm s^−1^ which is in keeping with the iron atom in the latter complexes being more electron rich and thus needing to withdraw less electron density from the porphyrin. This is in support of the findings from the visible spectra. In fact, the findings also agree well with previously reported data given in Table 4 where the average values of the isomer shifts for the TPP complexes is 0.24 mm s^−1^; for the PMXPP complexes, it is 0.27 mm s^−1^ and for the phthalocyanine (pc) complexes, it is 0.18 mm s^−1^ (all allowing for the temperature differences by adding 0.07 mm s^−1^ to room temperature data to bring it in line with the 78 °K data) [15–17, 66–76]. It thus appears that the different porphyrins contribute different amounts of electron density to the iron(II)).

The fact that all the [Fe(II)(Por)L(CO)] complexes have smaller *δ* mm s^−1^ values than the [Fe(II)(Por)L_2_] complexes is due to the fact that the CO ligands require more electron density to form complexes. This has been discussed previously by others [66, 67, 70] including Reimer et al. [66] who also observed that the Δ*E*_Q_ values of the [Fe(TPP)(pip)_2_] [Fe(TPP)(pip)(CO)] and [Fe(TPP)(CO)_2_] complexes decrease from 1.44 mm s^−1^ to 0.47 mm s^−1^ and 0.25 mm s^−1^, respectively, by the replacement of piperidine with CO. They interpreted the results in relation to a net decrease in the mean axial bond lengths. Thus, the axial bond length is shorter in [Fe(TPP)(CO)_2_] than that of Fe–N(piperidine). There are similarities between the many Mössbauer results of [Fe(TPP)] and [Fe(PPIX)] complexes. This sometimes allows the interpretation of the [Fe(PP1X)] data from the corresponding [Fe(TPP)] data and vice versa. The Δ*E*_Q_ values presented here for the [Fe(PPIX)(CO)L] complexes (where L = a nitrogenous ligand) are similar to those of the [Fe(TPP)(CO)L] derivatives.

### Isomer shifts (*δ* values)

Connor et al. [70] described the decrease in the isomer shift in the [Fe(TPP)(CO)L] compounds as arising from a decrease in the *d*-electron density on the iron atom due to strong *π*-bonding between Fe and CO and weak *σ*-bonding from the CO to the Fe. They went on to say that such a strong *π*-donation from iron metal to an occupied π* level of the porphyrin will decrease the population of the electron density on the Fe *d*_*xz*_, *d*_*yz*_, and *d*_*xy*_ orbitals. This will result in the s-orbital on the Fe being less shielded resulting in an increase in s-electron density at the nucleus. Although this appears to explain the decreasing value of the isomer shifts observed by them and those reported herein, others have pointed out that there are problems with this explanation; we will discuss the problems more fully in a follow-up paper.

**Fig. 3:**
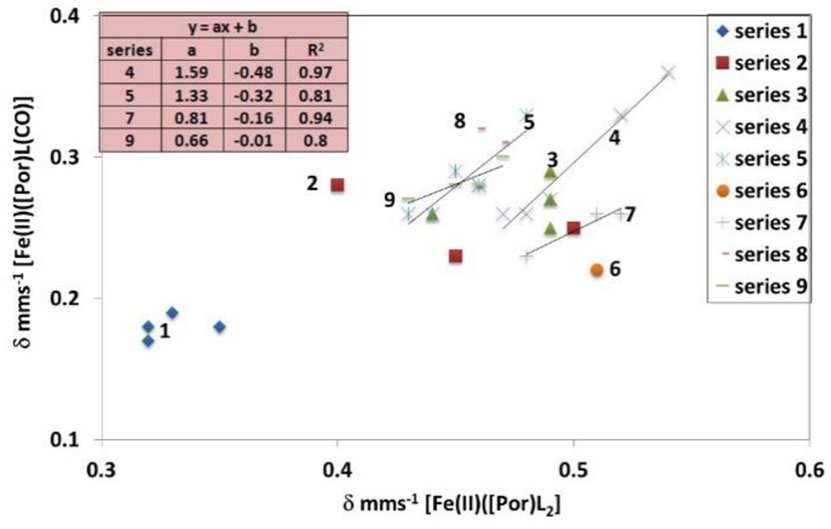
Plot of δ mm s^−1^ of the [Fe(II)(Por)L(CO)] complexes against the δ mm s^−1^ of the [Fe(II)(Por)L_2_] complexes. Series 1 = Pc complexes; Series 2 = TPP complexes; Series 3 = PMXPP complexes; Series 4 = PPIX (aliphatic nitrogenous) complexes; Series 5 = PPIX (aromatic nitrogenous) complexes; Series 6 = PPIX (2-MeIm) complex; Series 7 = PPIX (sterically hindered nitrogenous) complexes; Series 8 = TPPS (sterically hindered aliphatic complexes) complexes; Series 9 = TPPS (aromatic nitrogenous) complexes

In Fig. 3, a plot of *δ* values of the [Fe(II)(Por)L(CO)] complexes against the *δ* values of the [Fe(II)(Por)L_2_] complexes is presented for nearly all the complexes presented in Tables 3 and 4. Unfortunately, though we have 15 [Fe(II)(PPIX)L(CO)] complexes and 7 [Fe(II)(TPPS)L(CO)] complexes available from Table 3, in Table 4, there are fewer complexes that have literature values for both the [Fe(II)(Por)L(CO)] and [Fe(II)(Por)L_2_] complexes (in fact there are four [Fe(II)(Pc)L(CO)] complexes, three [Fe(II)(TPP)L(CO)] complexes, and four [Fe(II)(PMXPP)L(CO)] complexes).

We have included four trend lines in Fig. 3. The first is for Series 4 where there is a linear trend in the relationship between the *δ* of the [Fe(II)(PPIX)L(CO)] complexes against the *δ* of the [Fe(II)(PPPIX)L_2_] complexes, we can deduce that as a CO replaces the aliphatic L ligand, the change in the *δ* value is systematic with the nature of the aliphatic ligand (this is for five complexes). Similarly for Series 5, when L is an aromatic ligand, there is a similar trend of the [Fe(II)(PPIX)L(CO)] complexes against the *δ* of the [Fe(II)(PPIX)L_2_] complexes (in this case for six complexes). For the three sterically hindered aliphatic nitrogenous ligands [Fe(II)(PPIX)L(CO)] complexes (Series 7), there is again a linear relationship in the change in *δ* mm s^−1^ values, but this is different to the that of the non-sterically hindered of aliphatic complexes. Finally, for the remaining aromatic [Fe(II)(PPIX)L(CO)] complex 9 (Series 6 in Fig. 3), (where L = 2-MeIm), the complex plots amongst the aliphatic ligands and this is expected as the methyl group probably blocks the possibility of *π*-bonding, so the ligand becomes predominantly *σ*-bonding like the aliphatic ligands.

Four of the [Fe(II)(TPPS)L(CO)] complexes (Series 9) (where L = an aromatic nitrogenous ligand) manifest a linear trend and lie close to the [Fe(II)(PPIX)L(CO)] complexes (where L = an aromatic nitrogenous ligand). Of the other three [Fe(II)(TPPS)L(CO)] complexes (Series 8), two of the aliphatic pyrrolidine and piperidine ligand complexes also lie close to this trend line, whereas the complex containing the glycine ethyl ester lies on this trend line. Thus, all seven TPPS complexes lie close together. It is apparent that the TPPS complexes though showing a trend do not behave the same way as the PPIX complexes clearly demonstrating the two porphyrins have different bonding properties.

The three red squares in Fig. 3 are the [Fe(II)(TPP)L(CO)] complexes (Series 2). One of these (where L = piperidine) lies as expected in the mix of the aliphatic and sterically hindered nitrogenous ligands towards the bottom right of Fig. 3. The second complex (the pyridine complex) lies on the left of the aromatic complexes area. The third red square representing the position of 1-methylimidazole lies between the other two and below them; this is a good *σ*-donating ligand, and though it is aromatic, it is less sterically hindered than the pyridine type ligands and closer to the aliphatic ligands but below them.

Three of the [Fe(II)(PMXPP)L(CO)] complexes (Series 3) contain aliphatic ligands and lie close to the area aliphatic nitrogenous ligands of the PPIX, the fourth complex (pyridine) lies low on the left towards the bottom of the aromatic nitrogenous ligands. Therefore, it appears that all the complexes with aliphatic ligands lie in the same area (on the right of Fig. 3) for the iron porphyrins and the aromatic ligands are all in a separate area (on the left of Fig. 3).

The four [Fe(II)(Pc)L(CO)] complexes lie in a bunch on the extreme bottom left of Fig. 3. It is not surprising that these are well separated from the porphyrin complexes as the phthalocyanine macrocycles are good *σ*-donating ligands but are not good *π*-bonding ligands, so are expected to behave differently to the porphyrins.

There are three more complexes in Table 4 that have known Mössbauer parameters for the corresponding [Fe(II)(Por)L_2_] complexes that we have not actually plotted in Fig. 3. The first is the [Fe(TpivPP)(1-MeIm)(CO)] complex which would lie near the bottom of the aromatic complexes. The next is the [Fe(OMTBP)(pip)(CO)] which lies above the aromatic complexes on the left. This OMTBP complex would be expected to be very different as this is a much more extensive conjugated macrocycle, which is a good *π*-bonder but a poor *σ*-bonding ligand. The final complex is the only OEP complex, [Fe(OEP)(1-MeIm)(CO)], this lies between the aromatic and aliphatic regions towards the bottom of these compounds and as such is very close to the [Fe(II)TPP(1-MeIm)(CO)] complex.

From the above analysis presented in Fig. 3 and the data in Tables 3 and 4, it is apparent that for a given porphyrin, there is a linear trend in the relationship between the change in the *δ* mm s^−1^ value of the [Fe(II)(Por)L_2_] complexes on binding CO, which is related to the kind of ligand (whether aliphatic or aromatic). If the ligand is sterically hindered, this relationship is negated but the resulting position in Fig. 3 is still systematic. Even where there are not enough complexes for a given porphyrin the positions of the complexes is not random but follows the trends found for the PPIX and the TPPS complexes. It appears that each porphyrin can donate extra electron density to the iron(II) ion when a CO molecule replaces a nitrogenous ligand. The amount of electron density donated depends on the nature of the porphyrin and the fact that all the complexes do not lie on the same trend lines is evidence for each porphyrin being different in its ability to donate electron density. These finding are in keeping with the changes seen in the visible spectra section discussed above.

The Mössbauer spectra for complexes 8–10 of the [Fe(PPIX)(CO)L] complexes (Table 3) all contain some remaining parent [Fe(PPIX)L_2_] complexes; this suggests that these (CO) complexes are not as strong complexes as the others listed in Table 3 and this may be caused by the steric hindrance stopping the nitrogen ligands from getting closer to the porphyrin during the bond formation.

It is apparent from the trend lines in Fig. 3 that as the nitrogenous ligand binds more strongly to the Fe(II) atom in both the [Fe(II)(Por)L_2_] complexes and the [Fe(II)(Por)L(CO)] complexes then the position of the resulting point for a given porphyrin and nitrogenous ligand is closer to the bottom left of the plot. This is apparent in the Im complexes of Fe(II)(PPIX) and the 1-MeIm complexes of Fe(II)(TPP). This finding allows an important comparison to be made to the haemoglobin and myoglobin carbonyl complexes (see Table 4), both have *δ* values of about 0.2 mm s^−1^ at 4.2 °K. Taking this *δ* value and plotting it against the *δ* value of 0.42 mm s^−1^ (that we previously reported for [Fe(II)(PPIX)(histidine)_2_] [41]) in Fig. 3, would generate a point closest to the bottom left of the porphyrin complexes and also lie close to the line for Fe(II)(PPIX) indicating that the histidine residues binds very strongly to the Fe(II) cation. [It is important to note that in haemoglobin and myoglobin the axial ligand is a histidine residue and that the Fe(II)(PPIX) moieties in these globins are surrounded by apolar protein residues. In contrast, the [Fe(II)(PPIX)(histidine)_2_] [41] complex was studied in frozen solution at pH 12 which is a polar environment. It is not necessarily invalid to plot them against each other as most of the interactions of [Fe(II)(PPIX)(histidine)_2_] with their surrounding will be non-polar and would not be expected to influence the Mössbauer spectra as they would be relatively remote from the Fe(II) cation. Hence, we can conclude that the comparison to the other nitrogenous ligands in Fig. 3 shows that the histidine residue is very electron donating to the Fe(II)(PPIX) in both haemoglobin and myoglobin and by analogy this is why CO binds so strongly to these biological molecules.

### Quadrupole splittings (Δ*E*_Q_ values)

The Δ*E*_Q_ value is a measure of the asymmetry of the electric field gradient experienced (in the cases in this paper) by the iron(II) ion. This field is caused by the combined distribution of bonding and nearby electrons located on the ligands (as well as on the iron(II) atom itself). The smaller the Δ*E*_Q_ value the more symmetrical is the electric field around the iron(II) ion. In low-spin octahedral Fe(II) porphyrin complexes, the 6 electrons present on the iron fill the *d*_*xy*_, *d*_*xz*_ and *d*_*yz*_ orbitals. The observed Δ*E*_Q_ values are mainly generated by the imbalance in the electron densities in the *d*_*x*_^2^_*–y*_^2^ and *d*_*z*_^2^ orbitals. It has been observed that in the Mössbauer spectra of low-spin [Fe(II)(Por)L_2_] complexes, the electric field gradients have positive signs indicating that the covalent bonding to/from the 4 N atoms in the porphyrin to the Fe atom is stronger than that of the axial ligands [67]. This means that as the covalent bonding from the axial ligands increases then the Δ*E*_Q_ values decrease. Thus, replacing an L ligand with a CO generates a more symmetrical electric field.

**Fig. 4:**
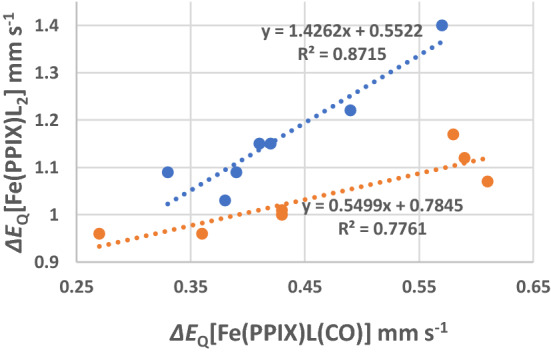
Plot of Δ*E*_Q_ values of [Fe(PPIX))CO)L] versus Δ*E*_Q_ values of [Fe(PPIX))L_2_]. The blue circles are the aliphatic ligands listed in Table 2. The red circles are the non-aliphatic ligands and refer to the 5-membered rings and sterically hindered ligands listed in Table 2. The trend lines are linear regression lines for the two indicated groups of ligands

The Δ*E*_Q_ values of the parent [Fe(PPIX)L_2_] complexes have been plotted versus those of the[Fe(PPIX)(CO)L] complexes for the same ligands in Fig. 4. The two trend lines in Fig. 4 show two interesting features: (1) the slopes differ substantially from each other and the values of the slopes, 0.55 and 1.43, respectively, differ significantly from 1. A slope of 1 would be expected if the effect of CO substitution would cause a constant shift of *π*-electron density in the axial direction. Apparently, this is not the case. It is to be noted that the error (see Table 3) on any value has a maximum of 0.02 mm s^−1^ so the linear trends are very reasonable.

The aliphatic ligands all have good *σ*-donating properties and hence donate their electrons to the iron to form single simple *σ*-bonds all these bonds would be of similar length except where steric repulsion is present for example in piperidine. These ligands do not form any *π*-bonds with the iron as they have no empty orbitals that can receive electrons. When one of these ligands is replaced by a CO, the iron axial bonding changes substantially concomitantly with the electron distribution along the axis. The CO forms a *σ*-bond directly to the iron but in addition it receives electrons into its empty antibonding *π*-orbitals (from the iron 3*d*_*xz*_ and 3*d*_*xz*_ orbitals) forming additional bonding to the iron and simultaneously this weakens the strength of the C–O bond.

The non-aliphatic ligands all have both a *σ*-bond and some *π*-bonding to the iron atom, when one of these ligands is replaced by a CO it competes for *π*-bonding with the other axial ligand. The fact that the slope of the trend line for the non-aliphatic ligands is different (from that of the aliphatic ligands) shows that the bonding strength of the CO to iron is different in the presence of these non-aliphatic ligands. In the following paragraphs, we shall elaborate on this interesting result.

The plot indicates that the aliphatic nitrogenous ligands lie on/straddle the same linear trend line, except for the sterically hindered ligands that lie on the other trend line along with the other five membered ring ligands. Imidazole in fact lies at the bottom on both lines.

The complexes of cystine methyl ester and glycine methyl ester, which are included in Fig. 1, also lie on the line with the non-sterically hindered ligands [27]. Figure 4 manifests the outlier position of piperidine (blue circle at top right-hand side of Fig. 1) in the group of aliphatic ligands. It is assumed that the electric field gradient at the iron(II)-atom is increased due to relatively large C–N–C angle of 110.7° at the bonding N atom in piperidine causing a small decrease of electron density in the Fe–N bond as compared to the other aliphatic ligands.

The relationship found in the plot supports the existence of a trans-effect in the [Fe(PPIX)(CO)L] complexes and this appears to be related to the nitrogenous ligand bonding strength. However, it is apparent that the fit of the data to the trend lines is not fully satisfactory, and it can be concluded that the trans affect is not uniform as the L ligand changes its bonding to the CO through the Fe atom even for the aliphatic nitrogenous axial ligands. This is evidence for steric effects in some of the axial ligands playing a role in interfering with the *σ*-bonding in the aliphatic ligands. The trend line for the five and six membered partially or completely aromatic nitrogenous ligands is not a very good correlation and reflects the fact that these ligands manifest both *σ*- and *π*-bonding to different extents. In a follow-up paper, we will return to this finding and illustrate how it can be expected/explained.

**Fig. 5:**
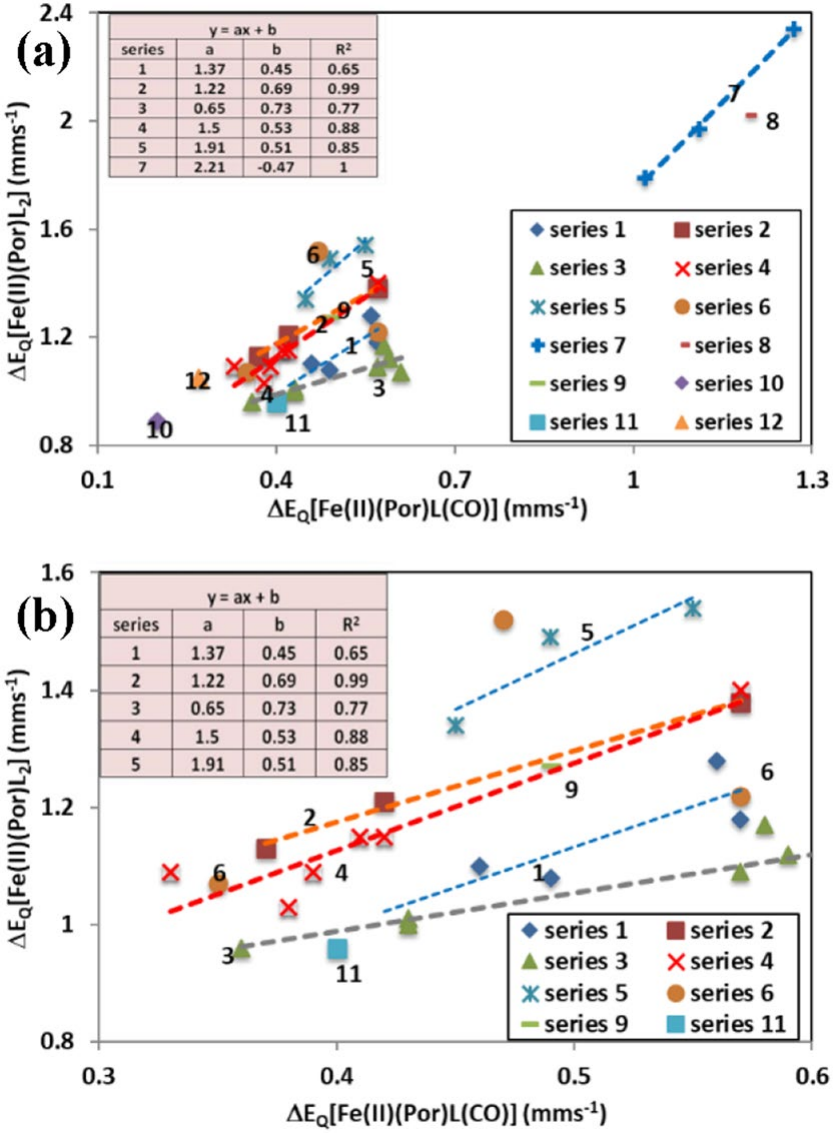
Plot of the Δ*E*_Q_ values (mm s^−1^) of the [Fe(II)(Por)L(CO)] complexes against the Δ*E*_Q_ values (mm s^−1^) of the [Fe(II)(Por)L_2_] complexes. Plot **a** shows all the series of complexes studied, whereas Plot **b** is an enlargement of the congested area of Plot a which allows the trend lines to be seen much more clearly. Series 1 = TPPS (aromatic nitrogenous ligand) complexes; Series 2 = TPPS (aliphatic nitrogenous ligand) complexes; Series 3 = PPIX (aromatic nitrogenous ligand) complexes; Series 4 = PPIX(aliphatic nitrogenous ligands) complexes; Series 5 = PMXPP (aliphatic nitrogenous ligand) complexes; Series 6 = 3 TPP complexes no trend line indicated because of low *R*^2^ value; Series 7 = Pc (aliphatic nitrogenous ligand complexes) complexes; Series 8 = the Pc pyridine complex; Series 9 = is a PMXPP pyridine complex Series 10 is the only OMTBP complex (1-MeIm); Series 11 is the solitary OEP 1-MeIm complex; Series 12 is the only TpivPP 1-MeIm complex

**Fig. 6:**
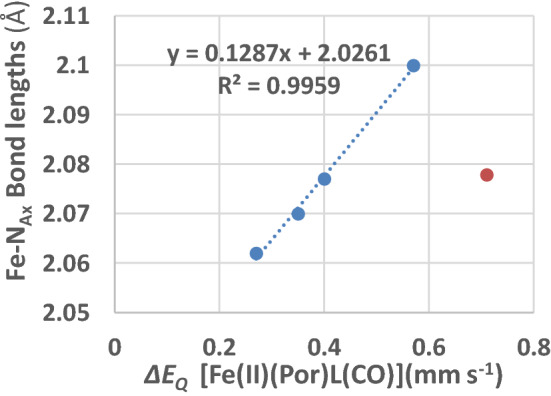
Plot of the Δ*E*_Q_ values of the [Fe(II)(Por)L(CO)] complexes against the Fe–N_Ax_ bond distances of the nitrogenous axial ligand. The blue circles are the data for [Fe(II)(TPP)L(CO)] (where L = 1-MeIm and py) as well as those for [Fe(II)(OEP)(1-MeIm)(CO)] and [Fe(II)(Tpiv_2_-C_12_P)(1-MeIm)(CO)]; the red circle is the data for [Fe(II)(TPP)(1,2-Me_2_Im)(CO)]

However, to show the trends are real and in fact all of the known [Fe(II)(Por)L(CO)] complexes (where the [Fe(II)(Por)L_2_] complexes for the L ligand are known), we present in Figs. 5 and 6 the [Fe(II)(TPPS)L(CO)] data from Table 3 and all the [Fe(II)(Por)L(CO)] data from Table 4 along with the data already presented in Fig. 4.

In Fig. 5, a plot of Δ*E*_Q_ of the [Fe(II)(Por)L(CO)] complexes against the Δ*E*_Q_ of the [Fe(II)(Por)L_2_] complexes listed in Tables 3 and 4 is presented. This plot contains 12 series of macrocyclic complexes. Ten of the series are porphyrin complexes and the other two are phthalocyanine complexes. Series 1 = TPPS (aromatic nitrogenous ligand) complexes; Series 2 = TPPS (aliphatic nitrogenous ligand) complexes; Series 3 = PPIX (aromatic nitrogenous ligand) complexes; Series 4 = PPIX (aliphatic nitrogenous ligands) complexes; Series 5 = PMXPP (aliphatic nitrogenous ligand) complexes; Series 6 = 3 TPP complexes. The first where the ligand is piperidine, which is close to the complexes in Series 5, the second where the ligand is pyridine is close to the ligands in Series 1, and the third where the ligand is 1-MeIm is in the region where the other Imidazole ligands are found near the bottom left of Series 4. Series 7 = Pc (aliphatic nitrogenous ligand complexes) complexes that are well removed from the porphyrin complexes due to the bonding properties of the Pc macrocycle; Series 8 = Pc the pyridine complex, this lies to the right of the trend line for the Pc complexes in Series 7 in accord with all the aromatic nitrogenous ligand complexes lying to the lest and below the aliphatic complexes; Series 9 = PMXPP pyridine complex which lies on the left of the three ligands in series and is tending towards the other pyridine complexes though it is not amongst them due to the properties of PMXPP; Series 10 is the only OMTBP complex (1-MeIm) and this lies close to the other Im and 1-MeIm complexes but is on the bottom left due to the bonding properties of OMTBP; Series 11 is the solitary OEP complex, it also contains a 1-MeIm ligand and lies just below the aromatic nitrogenous ligands in Series 3, not far from the other Im ligands; Series 12 is the only TpivP complex: this is also a 1-MeIm complex and lies close to those from series below the (aromatic nitrogenous) complexes.

It is clear from Fig. 5 that all the aliphatic nitrogenous ligand complexes lie towards the left of the plot and for the three Series 2, 4, and 5 the *R*^2^ values are 0.99 (for three complexes), 0.88 (for seven complexes) and 0.85 (for three complexes), respectively. Thus, 13 aliphatic nitrogenous ligand porphyrin complexes show similar trends. In addition, the aliphatic TPP piperidine complex lies close to them.

It is also apparent from Fig. 5 that the aromatic nitrogenous ligand complexes in Series 1 (four complexes, *R*^2^ value is 0.65) and Series 3 (eight complexes *R*^2^ value is 0.77) respectively, also manifest linear trends, Thus, 12 aromatic nitrogenous complexes show similar trends. In addition, the TPP pyridine complex also lies close to them showing all these aromatic nitrogenous ligands behave similarly in their porphyrin complexes and different to those of the aliphatic nitrogenous ligand complexes.

The Im and 1-MeIm ligand porphyrin complexes all behave similarly and locate between the above two groups, these ligands are good *σ*-bonding ligands and also manifest reasonable *π*-bonding properties and in addition exhibit little steric hindrance. The [Fe(II)(Por)COL] complexes containing these ligands manifest the smallest Δ*E*_Q_ values as the two axial ligands bind strongly.

It is now obvious from Figs. 3, 4 and 5 that each porphyrin lies on a separate line indicating that each porphyrin has different bonding properties. The closest to PPIX is in fact TPP, but they are not the same. The reason for this is that in Fe(II)(PPIX) complexes the vinyl and propionate groups can affect the bonding when they take up different positions relative to the porphyrin plane. In contrast in Fe(II)(TPP) complexes the four phenyl groups can rotate towards or away from the porphyrin plane.

Sams et al. [66] assumed that the porphyrin ring has electron sink capabilities in such a way that the iron complexes can modify their *σ*- and *π*-bonding characteristics to suit the functions in which metallo-porphyrins participate in biological systems. The data presented in Fig. 4 adds evidence in support of the electron sink capability for [Fe(PPIX)], where the effect of changing a nitrogenous ligand for a CO molecule changes the quadrupole splitting; however, from the discussion above the change is perhaps not as systematic as assumed by Sams et al. [66].

The following analysis is similar to that used for *δ* values in Fig. 3 though now it is applied to the Δ*E*_Q_ values in Fig. 5. From the trend lines in Fig. 5, it is obvious that as the nitrogenous ligand binds more strongly to the Fe(II) atom in both the [Fe(II)(Por)L_2_] complexes and the [Fe(II)(Por)L(CO)] complexes the Δ*E*_Q_ become smaller showing a more symmetric electronic environment around the Fe(II) cation. As they become more symmetric the position of the resulting point for a given porphyrin and nitrogenous ligand is closer to the bottom left of the plot just as found for the *δ* values in Fig. 3. This finding also allows a comparison to be made to the haemoglobin and myoglobin carbonyl complexes (see Table 4), both have Δ*E*_Q_ values of about 0.35 mm s^−1^ at 4.2 °K. This Δ*E*_Q_ value can be plotted against the Δ*E*_Q_ value of 0.88 mm s^−1^ we have previously recorded for [Fe(II)(PPIX)(histidine)_2_] in Fig. 5. In that case, it would be the closest point to the bottom left of the porphyrin complexes and lie close to the line for Fe(II)(PPIX). Here, we can see that the comparison to the other nitrogenous ligands in Fig. 5 shows that the histidine residue is very electron donating to the Fe(II)(PPIX) and is non-sterically hindered in both haemoglobin and myoglobin and that this again provides insight to why CO binds so strongly to these biological molecules.

### Molecular structural data

Having established above how the values of *δ* and Δ*E*_Q_ change as the axial nitrogenous ligand donate electron density to the Fe(II) in the [Fe(II)(Por)(CO)L] complexes, we now turn to the known crystallographic data (see Table 5) to see how they fit with our findings. The [Fe(II)(Por)L(CO)] complexes have been previously discussed by Scheidt et al. [69]; they reported structural and spectroscopic correlations that provide evidence for the *π* back-bonding model for CO bonding to the Fe(II)(Por)L moiety. Before discussing this and their other findings, we will first summarise the known molecular structures.

For six coordinate low-spin iron(II) porphyrin complexes, typical Fe–N (where N is an axial nitrogen ligand,) bond lengths depend on the nature of the nitrogen ligand as well as the nature of the other axial ligand. Thus, for aromatic ligands such as pyridine Fe–N = 2.10(1) Å in [Fe(TPP)(py)(CO)], 2.037(1) Å and 2.039(1) Å [68] in [Fe(TPP)(py)_2_] [85, 86] clearly showing that the pyridine bond length lengthens as the CO *π*-back bonds to the Fe(II)(Por)L moiety clearly indicating that the porphyrin alone cannot supply all the extra electron density needed by the CO. This finding is clearly repeated in the case of the substituted TPP porphyrin the (5,15-[2,2’-(dodecanediamido) diphenyl]:α,α-10,20-bis(*o*-pivaloylaminophenyl)porphyrin = Por) complex [Fe(Tpiv_2_C_12_P)(1-MeIm)(CO)] [91], the Fe–N_Im_ distance is 2.062(5) Å is long, whereas for 1-MeIm in [Fe(TPP)(1-MeIm)_2_] [87] Fe–N_Im_ = 2.014(5) Å. Shorter axial bonds are also apparent in [Fe(TPP)(1-VinylIm)_2_] [87, 88] Fe–N_Im_ = 2.004(2) Å and in [Fe(TPP)(1-BzLIm)_2_] [88] Fe–N_Im_ = 2.017(4). Even shorter Fe–N_Im_ axial bonds are found in [Fe(TpivPp)(1-MeIm)_2_] [90] Fe–N_Im_ = 1.9958(19) Å and 1.9921(18) Å and in [Fe(TpivPp)(1-VinylIm)_2_] [90], the Fe–N_Im_ distances are 1.9979(19) Å and 1.9866(19) Å. There are three other [Fe(II)(Por)(1-MeIm)(CO)] complexes in Table 5, (for two of these Por = TPP [69, 77] and the third is an OEP [69] complex), the Fe–N_Im_ distances are 2.071(2) Å, 2.0503(14) Å and 2.077(3) Å, respectively. All are much longer than the Fe–N_Im_ distances found in the [Fe(II)(Por)L_2_] complexes discussed above [87–90]. This again is evidence that in the presence of CO, the Por of the Fe(II)(Por)L moiety cannot supply all the extra electron density required by the CO. In all cases where a CO is present, the Fe–N_Im_ bond is lengthened and is presumably weaker. In the [Fe(II)(TPP)1,2-MeIm)(CO)] complex [69], the Fe–N_Im_ bond (2.0779(11) Å) is slightly longer than in the less sterically hindered 1-MeIm complexes and here the Fe–N_Por_ bond is 1.985(8) Å is shorter than in the 1-MeIm complexes showing the Por can donate even more electron density to the iron(II) to compensate for its *π* back bonding needs to the CO. In the latter case, the Fe–CO bond length (1.7537(15) Å is not one of the shortest in Table 5) perhaps indicating that the Fe–N_Im_ bond is not much weaker than in the none sterically hindered 1-MeIm complexes. The Fe–CO bond lengths in the six [Fe(II)(Por)L(CO)] complexes in Table 5 are all in the range 1.728(6) Å to 1.793(3) Å and these have been shown to correlate with the CO bond lengths [69].

There is only one example of a saturated axial ligand piperidine. For piperidine, the analogous distance of [Fe(TPP)(pip)_2_] [99] is 2.127(3) Å. The bond lengths to iron in [Fe(TPP)(L_2_)] (L = nitrogen ligand) order are as follows: 1R-Im < pyridine < piperidine. This is as expected as imidazole is the best *σ*-donor of the first two while piperidine, which is only able to *σ*-donate, is sterically hindered. Another example of a sterically hindered predominantly *σ* bonding ligand is aza-ferrocene in the [Fe(TFPP)(Fe(C_5_H_5_)(C_4_H_4_N))_2_] complex where the Fe–N_aza_ bond length (2.05(2) Å) is also long for a [Fe(II)(Por)L_2_] complex.

It is, therefore, apparent to this point that the known crystal structures support the bonding implications discussed herein. These small distances support our earlier findings [43] and those of this work, and it follows on from this that such distances are similar to those found in naturally occurring haem proteins and that this is the reason for the widespread use of histidine as axial ligands to iron porphyrins [38, 41]. We emphasise histidine as we have reported the Mössbauer parameters for the [Fe(II)(PPIX)L_2_] complexes where L = histidine and histidine type ligands (Δ*E*_Q_ values are in the range 0.88–1.04 mm s^−1^) [41] and where L = imidazole ligands (Δ*E*_Q_ values are in the range 0.95–1.04 mm s^−1^) [38] in a variety of different solvents. These values compare well with the reported Δ*E*_Q_ value of 1.04(3) mm s^−1^ for reduced cytochrome *b*_5_ [92].

We have also shown that depending on the nature of the bonding properties of the axial nitrogenous ligands in [Fe(II)(PPIX)L_2_] complexes the Δ*E*_Q_ values can vary from 0.88 to 1.40 mm s^−1^ [43, 44]. Scheidt et al. [69] have suggested that from their results on the structures and Δ*E*_Q_ values of their [Fe(II)(Por)L(CO)] complexes that the Δ*E*_Q_ values could be sensitive to the geometry around the iron atom. From the range of Δ*E*_Q_ values reported in Tables 3 and 4, this is indeed the case.

Further evidence in favour of this explanation is presented in Fig. 6. This is a plot of the Δ*E*_Q_ values (given in Table 4) of the [Fe(II)(Por)L(CO)] complexes against the Fe–N_Ax_ bond distances of the nitrogenous axial ligand (presented in Table 5). This plot gives insight into how the Fe–N_Ax_ bond length is affected by the axial nitrogen ligand bonding to the Fe(II)(Por)(CO) moiety. Four complexes shown as blue circles (see caption for Fig. 6) lie on the trend line which has an *R*^2^ value of 0.996, indicating a very good correlation between Fe–N_Ax_ bond lengths and the Δ*E*_Q_ values of the [Fe(II)(Por)L(CO)] complexes. Three of these four complexes contain a 1-MeIm ligand and a CO ligand thus the only difference between them is the porphyrin macrocycle; so, it can be implied that it is the latter that causes the differences as the porphyrin is changed. The fourth complex contains pyridine as the axial ligand, this complex has the largest Δ*E*_Q_ value and the largest Fe–N_Ax_ distance showing the pyridine differs in its bonding to the Fe(II)(TPP)(CO) moiety compared to the 1-MeIm ligand.

The compound [Fe(II)(TPP)(1,2-Me_2_Im)(CO)] is indicated by the red circle; this does not fit the correlation and it is the only complex with a sterically hindered nitrogenous ligand. Clearly, the steric hindrance exerted by the ligand causes the larger Δ*E*_Q_ value at the Fe(II) atom. This is because the more sterically hindered ligand is restricted from binding strongly to the Fe atom lowering the covalent electron density in the *d*_*z*_^2^ orbital and hence increasing the imbalance with the *d*_*x*_^2^_*–y*_^2^ orbital thereby giving rise to the larger Δ*E*_Q_ value.

**Fig. 7:**
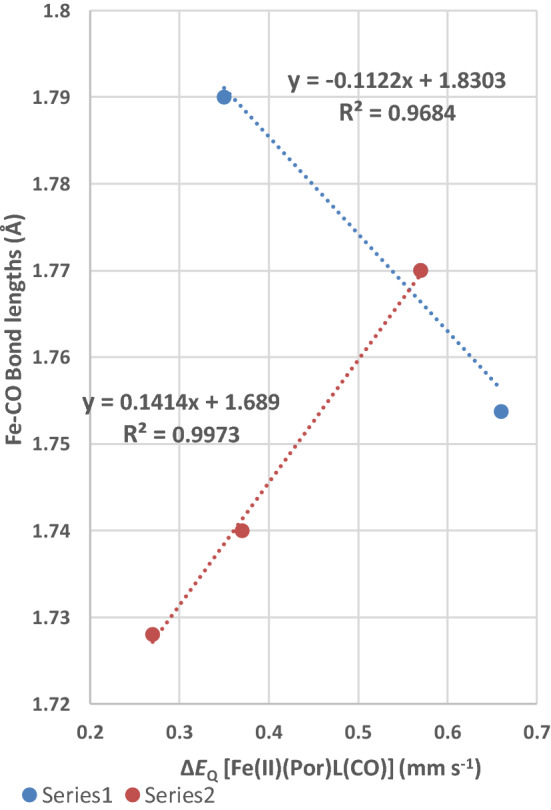
Plot of the Δ*E*_Q_ values of the [Fe(II)(Por)L(CO)] complexes against the Fe–CO bond lengths for each complex. Series 1 (three complexes) of [Fe(II)(TPP)L(CO)] (where L = 1-MeIm, py and 1,2-Me_2_Im); Series 2 (three complexes) two of these are [Fe(II)(Por)(1-MeIm)(CO)] complexes (where Por = Tpiv_2_-C_12_P and OEP) the third complex is the [Fe(II)(TPP)py(CO)] complex. The highest point of Series 2 (the [Fe(II)(TPP)(py)(CO)] complex) is included in the regression analysis of Series 1. The Δ*E*_Q_ values used are for the data closest to 77 °K

Figure 7 is a plot of the Δ*E*_Q_ values of the [Fe(II)(Por)L(CO)] complexes against the Fe–CO bond length of each complex. This plot gives insight into the asymmetry of the electric field from the viewpoint of the Fe–CO bond. There are five [Fe(II)(Por)L(CO)] complexes shown in the plot. The three points in Series 1 are the [Fe(II)(TPP)L(CO)] complexes (where L = 1-MeIm, py and 1,2-Me_2_Im, respectively), the trend line through these points gives an *R*^2^ value of 0.97, which is very good while it also has a negative slope. This negative slope shows that the Fe–CO bond length becomes smaller as the Δ*E*_Q_ value increases. As these three complexes all have the Fe(TPP) entity in common, their different Δ*E*_Q_ values must be primarily due to the different nitrogenous axial ligands. The fact the axial ligand bond lengths in these three complexes are all long and longer than in the [Fe(II)(TPP)L_2_] complexes (see Table 5) indicates that they are receiving less *π* back-bonding electron density from the iron(II) atom in the presence of the CO molecule which demands much more *π* back-bonding electron density from the iron(II) atom. It is important to realise that the Δ*E*_Q_ values relate to the asymmetry in the electronic field and that it is the change in this field that shows a linear trend with the Fe–CO bond lengths.

The three complexes in Series 2 are two [Fe(II)(Por)(1-MeIm)(CO)] complexes (where Por = Ppiv_2_-C_12_P and OEP) the third point is the [Fe(II)(TPP)py(CO)] complex which is also in Series 1. The trendline through these three points has an *R*^2^ value of 0.997, which is also a good fit to a straight line. This trendline has a positive slope indicating that as the Δ*E*_Q_ value increases so does the Fe–CO bond length. There is less in common in these three complexes in Series 2, each contains a different porphyrin two contain the same nitrogenous axial ligand and the third contains a py axial ligand. In comparison to Series 1, we might expect each porphyrin to manifest differing bonding properties to the iron (II) atom and its complexes to lie on a different possibly parallel line. If this assumption is correct, then the trendline for Series 2 would cross the three porphyrin lines and we can infer from this that changing the porphyrin will have a significant effect on the Δ*E*_Q_ value. The change in the Fe(II) bond length for the two 1-MeIm complexes is relatively small and could be interpreted as the two complexes have the different values simply because of the differing electron density distributions of the two porphyrins. In contrast the change in Fe–CO bond length moving up to the [Fe(II)(TPP)py(CO)] complex is much greater suggesting the change in nitrogenous axial ligand also effects the asymmetry of the electronic field around the Fe(II) atom and may be more important than the change caused by the porphyrin.

**Fig. 8:**
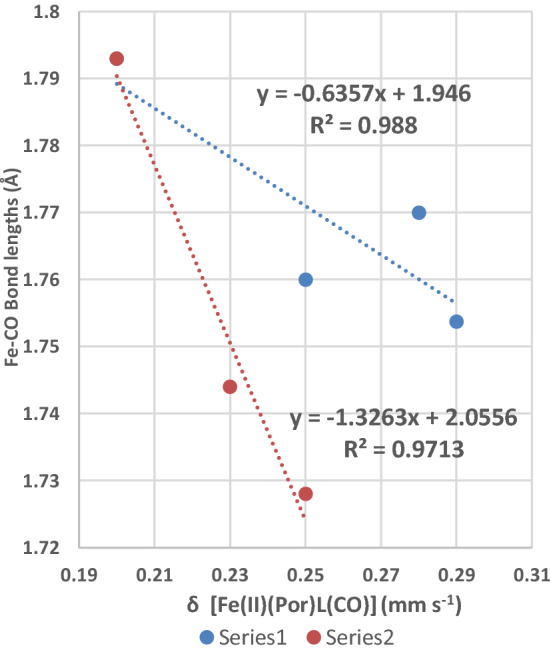
Plot of the δ values of the [Fe(II)(Por)L(CO)] complexes against the Fe–CO bond lengths for each complex. Series 1 (4 complexes) of [Fe(II)(TPP)L(CO)] (where L = 1-MeIm, py and 1,2-Me_2_Im and 1-MeIm where a solvent molecule is in the structure); Series 2 (3 complexes) 2 of these are [Fe(II)(Por)(1-MeIm)(CO)] complexes (where Por = Tpiv_2_-C_12_P and OEP) the third complex is the [Fe(II)(TPP)(1-MeIm)(CO)] complex. The highest point of Series 2 (the [Fe(II)(TPP)(1-MeIm)(CO)] complex) is included in the regression analysis of Series 1

The two trend lines fitted in Fig. 8 both have negative slopes indicating that as the δ values decrease the bond length of the Fe–C bond increases. Therefore, these two trend lines provide evidence for a relationship between the electron density on the Fe(II) cation (as observed at the Fe nucleus) in the porphyrin plane and the length of the Fe–C bond. As the Fe–C bond is made up of both *π*- and *σ*-bonding contributions from/to the Fe(II) cation, it is uncertain which is more involved.

**Fig. 9:**
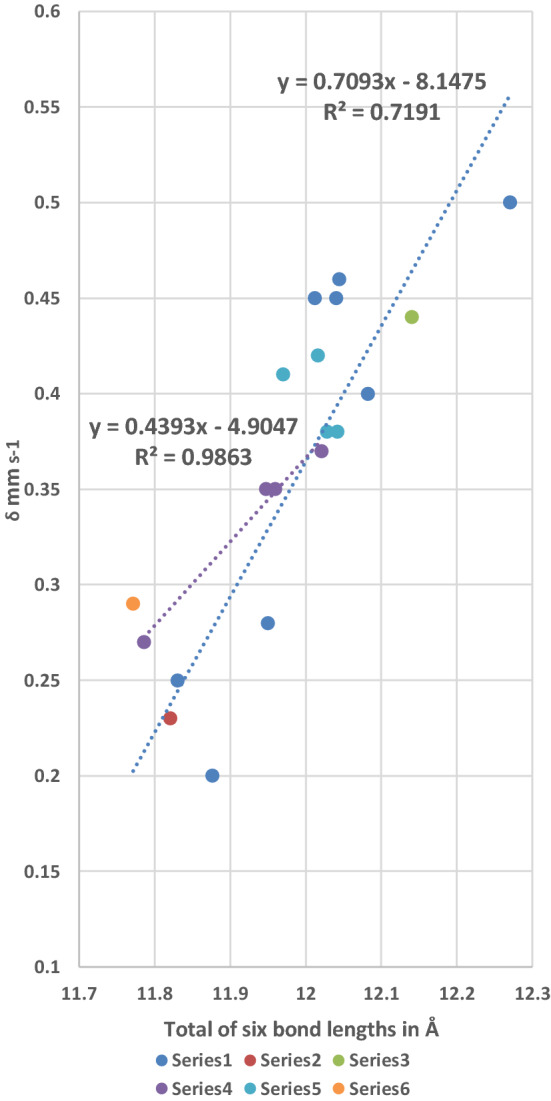
Plot of the total of six bond lengths around the Fe(II) cation in the [Fe(II)(Por)L_2_] and [Fe(II)(Por)L(CO)] complexes against the δ value (in mm s^−1^) of the complex. Series 1 eight points, Por = TPP; Series 2 one point, Por = OEP; Series 3 one point [Fe(TFPP)(Fe(C_5_H_5_)(C_4_H_4_N))_2_]; Series 4 four points Por = TpivPP; Series 5 four points Por = TMP; Series 6 Por = TPP, L = 1,2-MeIm

**Fig. 10:**
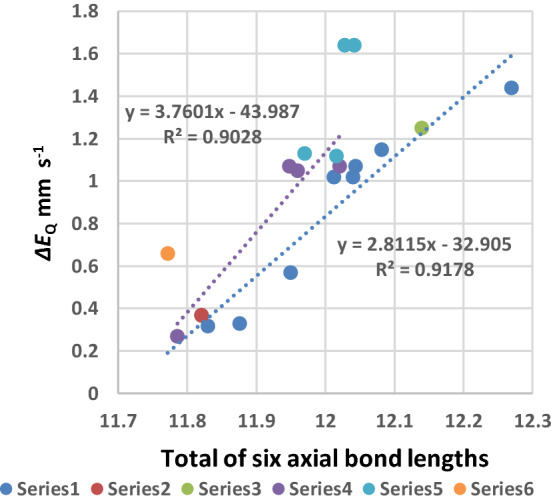
Plot of the total of six bond lengths around the Fe(II) cation in the [Fe(II)(Por)L_2_] and [Fe(II)(Por)L(CO)] complexes against the Δ*E*_Q_ value (in mm s^−1^) of the complex. Series 1 eight points, Por = TPP; Series 2 one point, Por = OEP; Series 3 one point [Fe(TFPP)(Fe(C_5_H_5_)(C_4_H_4_N))_2_]; Series 4 four points Por = TpivPP; Series 5 four points Por = TMP; Series 6 Por = TPP, L = 1,2-MeIm

Plots of *δ* against the Fe–N_axial ligand_ bond lengths and *δ* against Fe–N_porphyrin_ bond lengths do not manifest statistically significant trend lines and are, therefore, not presented. However, by considering the total six bond lengths that surround the Fe(II) cation in the [Fe(II)(Por)L(CO)] and {Fe(II)(Por)L_2_] complexes, two more plots have been constructed and are presented in Figs. 9 and 10.

Figure 9 presents a plot of the total of all six bond lengths (given in Table 5) around the Fe(II) cation of each complex against its *δ* value. Series 1 contains eight of the TPP complexes and the trend line through these points has an *R*^2^ value of 0.72, which is considered to be significant. However, the positions of the two points from Series 2 and Series 3 and the other trend line for Series 4 (which has four points and an *R*^2^ value of 0.99 (very good fit) all lie close to the trend line of Series 1. The four points in Series 5 also lie along this trend although these are all TMP complexes and two of them are from [Fe(II)(TMP)(2-MeHIm)_2_] complexes which contain sterically hindered ligands. The single point in Series 6 is the sterically hindered [Fe(II)(TPP)(1,2-MeIm)(CO)] complex and even this is not far from the trendline. Therefore, overall there is a trend seen in Fig. 9 that shows that as the total of the six bond lengths around the Fe(II) cation becomes smaller so does the δ value for both the [Fe(II)(Por)L_2_] and the [Fe(II)(Por)L(CO)] complexes. Thus, irrespective of whether the bonding in the bonds around the Fe(II) cations in the complexes is predominantly *σ* or *π* or a mixture of both, the total electron density as seen in the δ value is directly related to the distances of the six nearest neighbour bonding atoms.

Figure 10 presents a plot of the total of all six bond lengths (presented in Table 5) around the Fe(II) cation of each complex against its Δ*E*_Q_ value. Series 1 contains eight TPP complexes and the trend line through these points has an *R*^2^ value of 0.92 which is good. Series 4 contains the four TpivPP complexes and has a trend line with an *R*^2^ value of 0.90 (also significant). The single points in Series 2 and 3 are very close to the first trend line. The single point in Series 6 which is the [Fe(II)(TPP)(1,2-Me_2_Im)(CO) complex that contains the very sterically hindered 1,2-Me_2_Im ligand and two of the points of Series 5 the [Fe(II)(TMP)(2-MeHIm)_2_].

Complexes that contain sterically hindered ligands are the furthest points from the trend lines. Therefore, the total bond lengths are directly related to the Δ*E*_Q_ values. Thus, the smallest Δ*E*_Q_ values that are associated with the most symmetrical electronic environments are also directly associated with the smallest total bond lengths.

It is apparent from the relationships shown in Figs. 6, 7, 8, 9 and 10 and the information listed in Table 5 that the bond lengths of the ligands around the Fe(II) cations (from the axial ligands and the porphyrin nitrogen ligands) are all important in influencing the *δ* and Δ*E*_Q_ values. These findings can be explained by suggesting that when a CO binds to a Fe(II)(Por)L moiety or displaces the second L ligand in a [Fe(II)(Por)L_2_] both the porphyrin and the nitrogenous axial ligand modify their bonding to the Fe(II) cation. Moreover, as each different L ligand’s bonding is dependent on its outer orbitals and the presence or absence of any steric repulsions the porphyrin will need to be able to compensate its bonding to the Fe(II) cation donating more or less electron density as required. This finding is also evidence for the porphyrins having “electron sink” properties.

From the trend lines in Figs. 9 and 10 and the *δ* and Δ*E*_Q_ values for haemoglobin CO and myoglobin CO listed in Table 4, we would expect that the sum of the six bonds to the Fe(II) cations would be close to 11.8 Å in these proteins.

Comparing our prediction with three of the best refined crystal structures of myoglobin CO (that have structural resolutions of 1.5 Å, 1.2 Å, and 1.3 Å, respectively) we find the sum of the six bonds around the Fe(II) atom as 11.80 Å [100], 11.87 Å [101] and 11.82 Å [102]. Thus, the agreement with our result is exceptionally good especially as the four complexes in Fig. 10 have Δ*E*_Q_ values between 0.32 and 0.36 mm s^−1^ (see Table 4) and lie in the range 11.78 Å and 11.87 Å.

For haemoglobin CO, the protein is four times the size of myoglobin CO and the best refined crystal structures have much poorer final resolutions. However, there are three structures that seem very well refined: CO bovine Hb [103] (resolution 2.1 Å), R state CO human Hb [104] (resolution not given) and R2 state CO human Hb [105] (resolution 1.7 Å). The sums of the six bond lengths around the Fe(II) atoms differ for all three, viz.:—for the R2 state human Hb, there are four haems one in each subunit *α*1 = 11.85 Å, *α*2 = 12.03 Å, *β*1 = 11.87 Å, and *β*2 = 11.85 Å; for the R state CO human Hb, *α*1 = 11.9 Å; for *β*1 = 11.89; and for the CO bovine Hb, *α*1 = 11.90 Å, *α*2 = 11.91 Å, *β*1 = 12.03 Å, and *β*2 = 11.90 Å. In the case of the R state CO bovine Hb, three of the four haems have values close to 11.90 Å, for the R2 state CO human Hb, the two haems have values around 11.90 Å, while the R2 state human CO Hb has three haems around 11.85 Å and a fourth of 12.03 Å. Thus, the best resolved structure the R2 state of human CO Hb has three of its 4 haems in the range we predicted from Fig. 10 and the two haems of the R state of human CO Hb and three of the haems in CO bovine Hb have values of 11.90 Å which is just outside our predicted value range. We note that two haems have values of 12.03 Å which is outside our predictions, but we can say for CO Hb overall the agreement is very good.

### Biological significance of this work

Clearly, we have established that the smallest *δ* and Δ*E*_Q_ values for the {Fe(II)(Por)(CO)L] complexes were found for the L nitrogenous ligands that were the least sterically hindered. This was true whether the ligands were aliphatic or aromatic. Moreover, the smallest values were found for Im and 1-MeIm ligands that were both good *σ*- and *π*-electron donating. The histidine residue which is present as the axial nitrogenous ligand in both haemoglobin CO and myoglobin CO was shown using Figs. 3 and 5 to generate the smallest *δ* and Δ*E*_Q_ values. It was also possible to suggest bonding distances for the bonds round the Fe(II) cations in the proteins.

Clearly, the role of both haemoglobin and myoglobin is to bind oxygen reversibly; very early in the evolution of life on earth the histidine residue was favoured as the axial ligand to facilitate the oxygen binding. Unfortunately, what we have found evidence for in this work by studying a wide range of nitrogenous axial ligands is that histidine also facilitates the binding of CO. Luckily, there is not much CO in the natural environment for this to be a significant problem and even for individuals that smoke only a small percentage of the haemoglobin binds CO as the concentrations in the lungs of smokers is not too high.

In Table 4, we also list cytochrome P450_cam_CO which has a cysteine residue as the axial ligand as this enzyme needs to bind oxygen to detoxify metabolites in living systems, clearly this ligand also facilitates CO binding and manifests similar Mössbauer spectra when CO is bound. This indicates that CO binds tightly even in the presence of the cysteine residue just as in the presence of histidine in haemoglobin and myoglobin and has a dominant effect on the resulting Mössbauer spectroscopic parameters.

## Conclusions

It was shown herein that the behaviour of the visible spectra is very different for the [Fe(II)(PPIX)L_2_] complexes and the [Fe(II)(PPIX)L(CO)] complexes compared to those for the [Fe(II)TPPS)L_2_] complexes and the [Fe(II)(TPPS)L(CO)] complexes. From this, we can conclude that different porphyrins behave differently to one another depending on the electron charge they have available to donate to the Fe(II) atom. The fact that the spectra vary with the different nitrogenous ligands is evidence for the electron sink properties of the porphyrin moieties.

The [Fe(PPIX)(L)(CO)] complexes where L is a sterically hindered ligand were shown to form readily. The results on the three sterically hindered ligand complexes show that the presence of the CO stabilises/reinforces the bonding of sterically hindered ligands and is an indicator of how powerful CO bonding to [Fe(II)(Por)L] complex is.

The Mössbauer parameters given in Tables 3 and 4 were shown to be useful in gaining greater understanding on the bonding in the [Fe(PPIX)(L)(CO)] complexes. The isomer shifts of these complexes are reduced by about 0.2 mm s^−1^ from those of the corresponding [Fe(PPIX)(L_2_)] complexes, whilst the Δ*E*_Q_ values are reduced by around 0.6 mm s^−1^. The overall magnitude of the Δ*E*_Q_ though dominated by the contribution from the CO was shown to be affected by the bonding properties of the axial ligand and the porphyrin as well.

A novel approach to understanding changes in the bonding that take place at the iron(II) atom when a CO replaces a nitrogenous ligand is presented in the plots in Figs. 3, 4 and 5. These plots are (to our knowledge) original. Figure 3 a plot of the *δ* values of the [Fe(II)(Por)L(CO)] complexes against the *δ* values of the [Fe(II)(Por)L_2_] complexes (for nearly all the complexes presented in Tables 3 and 4) indicates that for a given porphyrin there is a linear trend in the relationship between the change in the *δ* value of the [Fe(II)(Por)L_2_] complexes on binding CO which is related to the kind of ligand (whether aliphatic or aromatic). If the ligand is sterically hindered, this relationship is negated but the resulting position in Fig. 3 is still systematic.

It appears that each porphyrin can donate extra negative charge to the iron(II) ion when a CO molecule replaces a nitrogenous ligand. The amount of electron charge donated depends on the nature of the porphyrin and the fact that all the complexes do not lie on the same trend lines is evidence for each porphyrin being different in its ability to donate electronic charge. These finding are in keeping with the changes seen in the visible spectra.

It is apparent that in Fig. 4 the nitrogenous aliphatic ligands lie on/straddle the same trend line and the non-aliphatic ligands lie on the other trend line. This finding indicates that CO bonds differently to the iron(II) atom depending on the nature/bonding properties of the other axial ligand. The relationships found in the plot supports the existence of a trans-effect in the bonding of the axial ligands in the [Fe(II)(PPIX)(CO)L] complexes and this appears to be related to the axial ligand bonding strength.

Figure 5 presents plots of the Δ*E*_Q_ values of the [Fe(II)(Por)L(CO)] complexes against the Δ*E*_Q_ values of the [Fe(II)(Por)L_2_] complexes for 12 series of complexes. The trend lines found in Fig. 5 reinforce those in Fig. 4 indicating that the relationships found for the binding of the axial ligands in the [Fe(II)(Por)(CO)L] complexes are reproducible. They only differ slightly from one porphyrin to another due to variation in bonding properties of the N-ligating ligands of each porphyrin. This clearly indicates that the electron density available for bonding in the different porphyrin cores varies from one porphyrin to another. It was found herein that the closest Fe(II)porphyrin (in terms of bonding properties) to Fe(II)(PPIX) in these [Fe(II)(Por)(CO)L] complexes is Fe(II)(TPP) but the bonding properties are not the same.

Figures 6, 7, 8, 9 and 10 show how the bond lengths in the known crystal structures of the [Fe(II)(Por)L_2_] and [Fe(II)(Por)(CO)L] complexes can be related to their Mössbauer parameters and how these findings back the relationships we presented in Figs. 3, 4 and 5. This approach enabled a greater understanding of factors that affect the strength of the bonding in haemoglobin CO and myoglobin CO. Finally, we were able to make predictions of the total of the six bond lengths around the Fe(II) cations in these protein complexes.
